# Identification of inhibitors targeting the energy-coupling factor (ECF) transporters

**DOI:** 10.1038/s42003-023-05555-x

**Published:** 2023-11-20

**Authors:** Eleonora Diamanti, Paulo C. T. Souza, Inda Setyawati, Spyridon Bousis, Leticia Monjas, Lotteke J.Y.M. Swier, Atanaz Shams, Aleksei Tsarenko, Weronika K. Stanek, Manuel Jäger, Siewert J. Marrink, Dirk J. Slotboom, Anna K. H. Hirsch

**Affiliations:** 1 Helmholtz Institute for Pharmaceutical Research (HIPS) − Helmholtz Centre for Infection Research (HZI), Campus Building E 8.1, D-66123 Saarbrücken, Germany; 2 Molecular Microbiology and Structural Biochemistry, UMR 5086 CNRS and University of Lyon, Lyon, France; 3 Laboratoire de Biologie et Modélisation de la Cellule (UMR 5239, Inserm, U1293) and Centre Blaise Pascal, École Normale Supérieure de Lyon, Université Claude Bernard Lyon 1 and CNRS, 46 Allée d’Italie, 69007 Lyon, France; 4 Biomolecular Sciences and Biotechnology Institute University of Groningen Nijenborgh 4, 9747AG Groningen, The Netherlands; 5 Department of Biochemistry, Bogor Agricultural University, Dramaga, 16680 Bogor, Indonesia; 6 Department of Pharmacy, Saarland University, Campus Building E8.1, 66123 Saarbrücken, Germany; 7 Stratingh Institute for Chemistry, University of Groningen, Nijenborgh 7, NL-9747 AG Groningen, the Netherlands

**Keywords:** Molecular modelling, Bacterial infection

## Abstract

The energy-coupling factor (ECF) transporters are a family of transmembrane proteins involved in the uptake of vitamins in a wide range of bacteria. Inhibition of the activity of these proteins could reduce the viability of pathogens that depend on vitamin uptake. The central role of vitamin transport in the metabolism of bacteria and absence from humans make the ECF transporters an attractive target for inhibition with selective chemical probes. Here, we report on the identification of a promising class of inhibitors of the ECF transporters. We used coarse-grained molecular dynamics simulations on *Lactobacillus delbrueckii* ECF-FolT2 and ECF-PanT to profile the binding mode and mechanism of inhibition of this novel chemotype. The results corroborate the postulated mechanism of transport and pave the way for further drug-discovery efforts.

## Abstract (web summary)

Virtual screening, biochemical assays, and coarse-grained simulations identify an inhibitor of energy-coupling factor transporters and its potential mechanism of action.

## Introduction

Energy-coupling factor (ECF) transporters are a recently discovered subclass of the superfamily of adenosine 5’-triphosphate (ATP)-binding cassette (ABC) transporters. While ABC transporters are present in all prokaryotic and eukaryotic species^1–4^, mediating uptake or extrusion of compounds into and from cells, the ECF transporters are absent in humans and other eukaryotes, with the notable exception of plant chloroplasts, but present in ~50% of prokaryotic species^5^. This class of proteins mediates the uptake of essential micronutrients such as water-soluble vitamins (e.g., folate^6^, riboflavin^7^, cobalamin^8^, biotin^9^, niacin^10^, thiamine^11^, pantothenate^12^) and metal cations (Ni^2+^ and Co^2+^)^13,14^ into bacteria and archaea. They are widely distributed in the *Firmicutes* phylum of Gram-positive species^15^ and contribute to the survival and growth of the bacteria^16^.

**Fig. 1:**
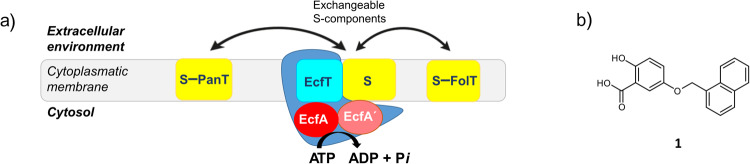
ECF group-II transporters and compound **1**. **a** Architecture of ECF group-II transporters. In yellow the S-components (e.g., the ones specific for folic acid (FolT) and panthotenate (PanT)), in red EcfA, in pink EcfA´ and in light blue EcfT, grouped in blue is the ECF module (EcfT, EcfA and EcfA´). The S-components interact with a shared ECF module. **b** Chemical structure of compound **1** identified through structure-based virtual screening using the crystal structure of *Lactobacillus delbrueckii* ECF-FolT2 (PDB ID 5JSZ)^29^.

The ECF transporters are transmembrane protein complexes consisting of two modules: an integral membrane protein dedicated to the binding of a transported substrate with high affinity and specificity (the S-component), an ECF module formed by an integral membrane protein, the T-component (EcfT) and two intracellular ATPases (EcfA and EcfA’, Fig. 1a). ECF transporters are classified into two groups, group I and group II^17^. In group I, the ECF module interacts exclusively with a single “dedicated” S-component, whereas the module in group II interacts with different ones. In group II, the same ECF module can associate with distinct S-components, opening up the possibility of blocking the uptake of several vitamins with a single inhibitor.

A recently proposed mechanism of transport postulates that the S-component can dissociate from the ECF module, which allows for exposure of the substrate binding pocket to the extracellular environment^18,19^. After binding, the substrate is buried inside a cavity, inducing a conformational change of S-component to its closed state. In this conformation, the S-component can rotate (topple over) by ~90 degrees in the membrane. In the toppled state, the S-component binds to the ECF module, which leads to the release of the substrate inside the cytosol^20^. Subsequent pinching of two long alpha-helical motifs, the coupling helices, of the EcfT subunit is predicted to occur by binding of ATP, and should cause re-orientation and dissociation of the substrate-free S-component, marking the end of a transport cycle. Hence, inhibitors that specifically block the dissociation of the S-component (as a molecular glue^21^) or the movements taking place in the ECF module, in particular the coupling helices, could impact the uptake of multiple vitamins (mediated by different S-components) by the bacterium. A more thorough investigation of both the mechanism of transport and the exploration of the potentially strategic binding pockets calls for the discovery of suitable tool compounds. With this idea in mind, our hit-identification campaign aimed to identify compounds acting as probes that can be used to study the mechanism of action.

Ultimately, as the inhibition of ECF transporters likely will affect growth and survival, it constitutes a potential avenue for intervention in bacterial infections. Despite the important functions of ECF transporters, however, so far only substrate-mimicking inhibitors for the S-component ThiT from *Lactococcus lactis* have been reported^22–24^, as well as two families of ECF inhibitors but with limitations in their utility for further studies^25,26^.

Here, we describe the discovery of compound **1** (Fig. 1b), as a promising ECF inhibitor potentially acting as protein-protein interaction (PPI) modulator^27,28^. We experimentally show that this compound is able to inhibit the uptake of more than one vitamin and may be used as a chemical probe to explore the function of ECF transporters in vitro.

## Results and discussion

### Hit identification

We performed structure-based virtual screening (SBVS) using the crystal structure of a folate-specific transporter ECF-FolT from *Lactobacillus delbrueckii* (PDB ID: 5JSZ)^29^. We chose this hit-identification strategy considering that for this protein a robust, albeit low-throughput activity assay is available^29^ and that the availability of crystal and cryo-EM structures might help to rationalize the binding mode of our novel chemical structures. In the SBVS, we used the algorithm DoGSiteScorer (Supplementary Notes 1, Supplementary Figs. 1 and 2)^30^ to select druggable pockets and, at the outset, we deprioritized the substrate- and ATP-binding pockets, because we were primarily interested in allosteric inhibitors. Therefore, we initially selected the so-called P2 pocket as the most promising allosteric binding site; it is in a location near the predicted surface of the membrane, where the EcfT component may change the conformation of two so-called coupling helices during transport (Supplementary Figs. 1 and 2). Compounds binding to this pocket may interfere with the transport cycle and simultaneously inhibit the uptake of multiple substrates. We then used the Express Collection of Princeton BioMolecular Research (1.3 million compounds) in combination with the KNIME Analytics Platform^31^ and LeadIT^26^ with the scoring function HYDE implemented in SeeSAR to perform the SBVS^27^. We followed a typical workflow (details are provided in Supplementary Notes 2), applying a range of filters to focus on drug-like compounds and exclude frequent hitters^32,33^. The final selection of the 100 top-ranked compounds included visual inspection, assessment of the docked pose, and estimation of the drug-like properties. As a result, we selected twelve distinct molecular scaffolds (**1**–**12**, Fig. 1 and Supplementary Fig. 3) for biochemical screening, of which only half were sufficiently water-soluble (Supplementary Fig. 4). Compounds **1** and **2** have been chosen as hits from this SBVS campaign^25^ (Supplementary Fig. 5). Hit **2** suffers from solubility issues given its *zwitterionic* nature; as a result, we selected Hit **1** for our study because of its chemical tractability and more favorable physicochemical properties.

### Biochemical assay to validate HIT 1

**Fig. 2:**
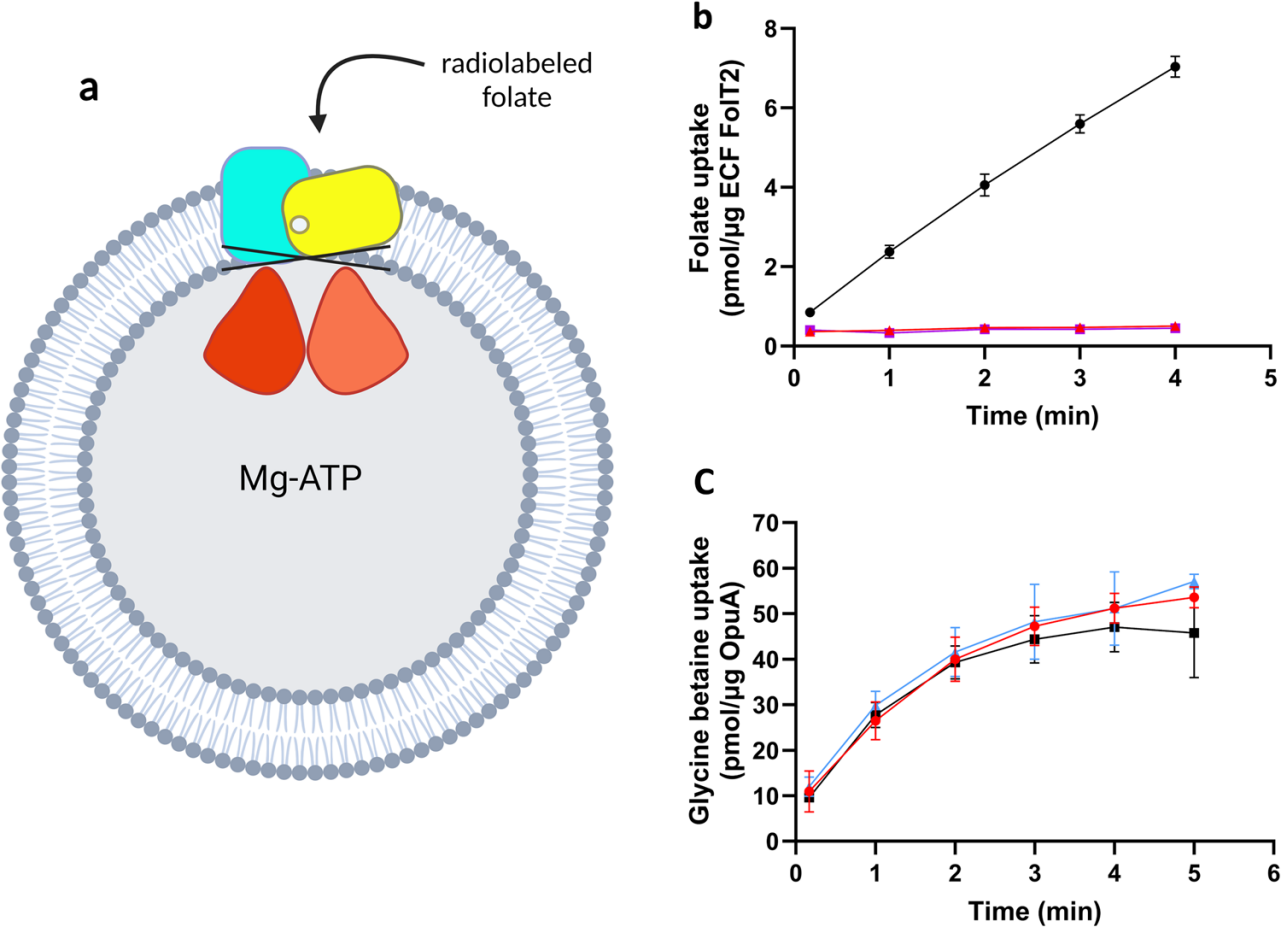
Effects of compounds 1 and 2 on the transport activity of ECF transporters ECF-FolT2 and an unrelated ABC transporter (OpuA). **a** Schematic representation of transport-activity assay. **b** Folate uptake by ECF-FolT2 reconstituted into proteoliposomes filled with 5 mM MgATP (black), 5 mM MgADP (purple), or 5 mM MgATP plus 5 mM of compound 1 (red), with a final DMSO concentration of 10% (v/v) in all experiments. **c** Glycine betaine uptake by OpuA in proteoliposomes filled with 5 mM MgATP (black), plus 5 mM of compound 1 (red). The blue curve represents proteoliposomes filled with 5 mM MgATP and no DMSO. The error bars represent the standard deviation of three independent measurements, except for the data in panel **c**, where they represent the spread of the data from two independent measurements.

A transport-activity assay on purified ECF-FolT2 reconstituted in proteoliposomes using radiolabeled folate showed that **1** is able to reduce translocation of the vitamin across the membrane with an IC_50_ value of 282 μM (Supplementary Notes 3, Supplementary Fig. 5) and we selected this compound for our studies (Fig. 2).

At an inhibitor concentration of 5 mM, compound **1** was able to completely inhibit the transport of folate by ECF-FolT2 (Fig. 2b). Next, we set out to validate that the inhibitory activity of **1** is not due to an artifact-like disruption of the lipid bilayer or chelation of Mg^2+^, which is crucial for the hydrolysis of ATP, or direct interference with the hydrolysis of ATP. Thus, we used the ABC transporter OpuA from *L. lactis*, a classical ABC transporter that mediates the uptake of glycine betaine, with membrane domains unrelated to ECF transporters, as negative control (Fig. 2c). Since the ATP binding sites are well-conserved among all ABC transporters, OpuA can be used to evaluate the selectivity of the compounds. OpuA was purified and reconstituted into liposomes. The uptake of glycine-betaine into the proteoliposomes was not affected by the presence of **1**, providing the first indication that our compound neither inhibits the hydrolysis of ATP by ABC transporters nor disrupts the lipid bilayer of the proteoliposomes or interferes with the accessibility of magnesium. In conclusion, we demonstrated in this step, that compound **1** is able to inhibit the transport of folic acid and selectively inhibit the family of ECF transporters but not classical ABC transporters in general.

### Searching for evidences of the mechanism of action

After demonstrating that hit **1** interferes with the uptake of folic acid via inhibition of the ECF transporter in vitro (Fig. 2), we looked to better understand its mechanism of action, and to do so we aimed to (i) validate the predicted binding pocket and to (ii) investigate the binding mode of **1** in ECF-FolT2.

As ECF transporters have a highly dynamic mechanism of function, traditional pocket predictions based on the analysis of single crystal or cryo-EM structures are not ideal. Therefore, we decided to perform coarse-grained (CG) molecular dynamics (MD) simulations based on the recently developed unbiased sampling approach^34^ using the Martini 3 force-field^35^ (more details in Supplementary Notes 5). This method has so far accurately predicted binding pockets and binding modes for pharmaceutically relevant targets such as nuclear receptors, GPCRs, and kinases. Predictions of binding affinities are also possible, in case of sufficient sampling^34,36–38^. The bacterial membrane model, the ECF-FolT2 transporter, and compound **1** were included in the system, which was simulated for a total of 0.3 milliseconds, allowing not only to explore all possible pockets but also to capture competition with the lipids for occupying protein pockets and enough association and dissociation events to estimate binding free energies (Δ*G*_bind_). For transmembrane proteins, the dynamics and the lipid environment are crucial components that need to be considered but are usually neglected in docking approaches. We predicted the binding of compound **1** to ECF-FolT2 using the apo structures of the folate-specific ECF transporter (ECF-FolT2)^39,40^.

**Fig. 3:**
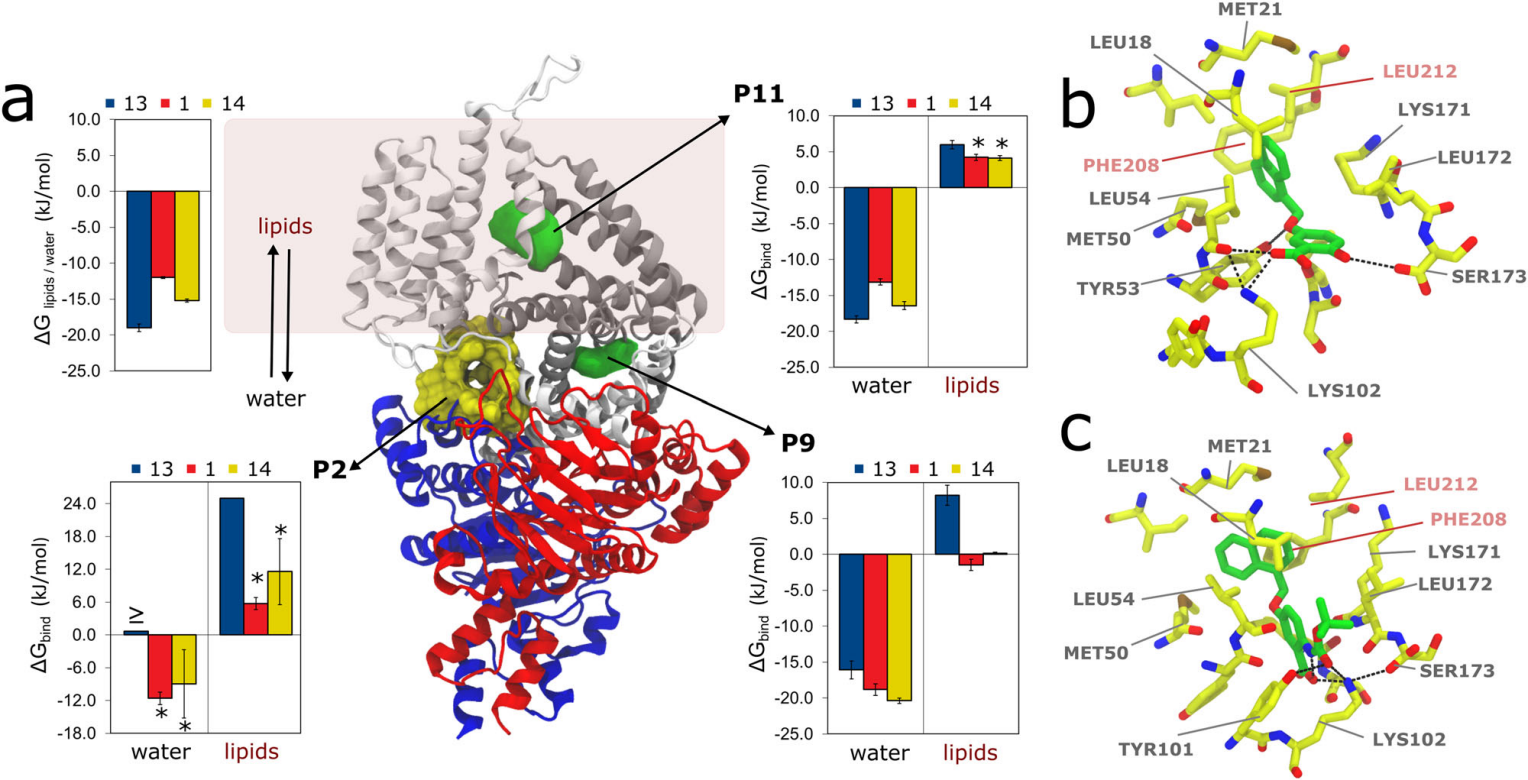
Unbiased coarse-grained (CG) molecular dynamics simulations of inhibitors binding to ECF transporter. **a** Crystal structure of ECF FolT2 (PDB ID 5JSZ), in complex with EcfA and EcfA’ shown in blue and red, EcfT in white, and S-component in gray. Pocket P2, used in the structure-based virtual screening campaign is highlighted in yellow. Compound **1** densities obtained from the simulations are shown in green, which indicates the existence of two other pockets, P9 and the entrance of P11 (pocket names according to Supplementary Fig. 1). The green isosurfaces correspond to regions with high occupancy (100 times higher than compound 1 density in the membrane). As compound 1 binds only with low affinity to P2, no high occupancy density is observed. The figure also shows bar plots with calculated binding free energies (Δ*G*_bind_) of compounds **13** and **14** for pockets P2, P9, and P11 in relation to water and membrane. In the case of compound 13 in pocket P2, the binding events are so reduced that it is only possible to estimate the upper limit (indicated by “≥” in the plots). For compound **14**, as the affinity for P2 is very low, the number of frames in which the ligand stays bound is low compared to the other binding sites. As a consequence, the error bars are larger in this case. Estimates of the partitioning free energies of the inhibitors between water and lipids (Δ*G*_lipids/water_) are also shown in the figure. The error bars represent the mean absolute error obtained by the block-averaging approach. **b**, **c** Representative poses of compounds 1 and 14 inside pocket P9, respectively, backmapped from the CG to the atomistic resolution. Some residues from EcfT (salmon) and S–component (gray) are highlighted, including the hydrogen-bond network (black dashed lines). Hydrogen atoms have been omitted for clarity.

The results for ECF-FolT2 indicate that compound **1** not only can bind in pocket P2, but also in two additional pockets: P9, which is a narrow, hydrophobic, and partially hidden pocket at the interface between EcfT and the S-component, and in the entrance of P11, which is the main cavity of the S-component where transported substrates pass to reach the position that they must occupy for transport (Fig. 3a). Although the binding of compound **1** to P2 is not so favorable, a negative Δ*G*_bind_ in relation to water (Δ*G*_P2-water_ = –11.6 ± 1.1 kJ/mol) may explain this compound being found as a hit in the SBVS campaign. Prediction of Δ*G*_bind_ in relation to water or membrane places P9 as the most probable binding pocket of ECF-FolT, with compound **1** showing a Δ*G*_P9–water_ and Δ*G*_P9–memb_ –18.8 ± 0.8 and –1.5 ± 0.8 kJ/mol, respectively. Importantly, as P2, the binding to P9 is also in line with the strategy of blocking the movements of the coupling helices taking place in the ECF module that are probably involved in the toppling and/or dissociation of the S-component. The entrance of P11 showed a stronger binding of compound **1** in relation to water than P2, with a Δ*G*_P11-water_ of –13.1 ± 0.4 kJ/mol. However, the binding is weaker than observed for P9. Additionally, the competition with the lipids for this pocket and/or the preferential partitioning of compound **1** to the membrane also makes this pocket less relevant, as P11 is embedded in the membrane environment. Indeed compound **1** can easily escape as the Δ*G*_P11-memb_ is 4.2 ± 0.4 kJ/mol. See Supplementary Fig. 14 and Supplementary Table 1 for more details regarding the free energy estimates.

**Fig. 4:**
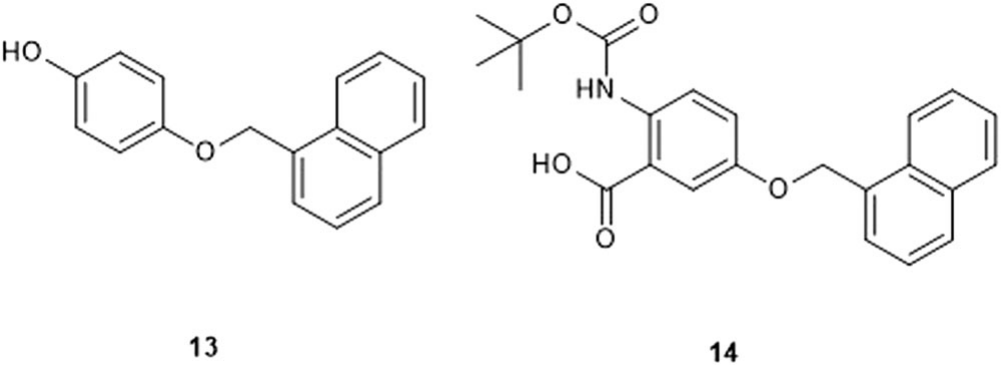
Chemical structure of compounds **13** and **14**. Representation of the chemical structures related to compounds **13** and **14** that have been used to support the predicted binding mode of hit **1**.

To further corroborate the predicted binding mode and the correlation between the in vitro results and MD studies, we designed and synthesized **13** and **14** (Fig. 4, synthesis on Supplementary Notes 4). Both compounds were designed to check the possible importance of the salicylic acid moiety in the interactions with ECF-FolT.

The removal of the carboxylic group (compound **13**) led to a loss of inhibitory activity, supporting the key role played by this group, while the replacement of OH by NHBoc led to increased potency (IC_50_ = 134 ± 26 μM). Comparison with additional results of CG molecular dynamics simulations corroborates these trends, reinforcing the hypothesis that P9 is the most probable pocket (Table 1 and Fig. 3). Δ*G*_P9–water_ estimated for compounds **1,** **13**, and **14** nicely correlate with the inhibitory activity, while for P11 compound **13** emerges with the highest binding affinity in relation to water (Fig. 3a). The proximity of P9 to the membrane and the highly favorable partitioning of the compound to the membrane may also explain the lack of inhibitory activity of **13** as its Δ*G*_P9–memb_ is unfavorable (+8.2 ± 1.41 kJ/mol), while compounds **1** and **14** show almost zero Δ*G*_bind_ for P9 in relation to the membrane (Supplementary Fig. 14 and Supplementary Table 1). As indicated by the lipids/water partitioning free energy (Fig. 3a), all compounds simulated tend to be located in the membrane. However, the removal of the carboxylate group (in relation to compound **1**) makes compound **13** too hydrophobic. The replacement of the hydroxyl group in compound **1** (Fig. 3b) by an NHBoc group (Fig. 3c) in compound **14** promotes additional hydrophobic interactions of the *tert*-butyl group with Leu 172. At the same time, the hydrogen bond of the OH group is maintained by the NHBoc group of compound **14**. These combined effects possibly explain the higher activity of **14**. As anticipated, the rigid molecular docking approaches, which disregard protein flexibility and the membrane environment, may fail to accurately predict the affinity trends observed in the experiments (see Supplementary Notes 6, Supplementary Fig. 16, and Supplementary Table 3).

**Table 1:** Inhibitory potencies (IC_50_ values and percentages of inhibition) in *Lactibacillus delbrueckii* ECF-FolT2 uptake assay; calculated Δ*G*_P9-memb_ (kJ/mol) and Δ*G*_P9-water_ (kJ/mol) of compound **1**, **13**, and **14** on ECF FolT-2 obtained from the MD simulations.

Cmpd	%Inh.@250μM ±S.E.M.^a^	IC_50_ (μM) ^b^	Δ*G*_P9-memb_ (kJ/mol)^c^	Δ*G*_P9-water_ (kJ/mol)^c^
1	34 ± 9	282 ± 108	−1.5 ± 0.8	−18.8 ± 0.8
13	No inhibition	-	8.2 ± 1.4	−16.1 ± 1.2
14	76 ± 4	134 ± 26	0.2 ± 0.1	−20.4 ± 0.4

^a^A total of three independent measurements were performed for every time points. The error bar represent the mean of sample standard deviation.

^b^A total of three independent measurements was performed for every time point and different concentration of compound. The error bar represents the mean of the sample standard deviation.

^c^A total of 10 MD simulation replicas were performed, with each production simulation carried out for 30 µs. The error bars represent the mean absolute error obtained by block averaging approach, with the total 300 µs sampling obtained by each compound divided in three blocks.

**Fig. 5:**
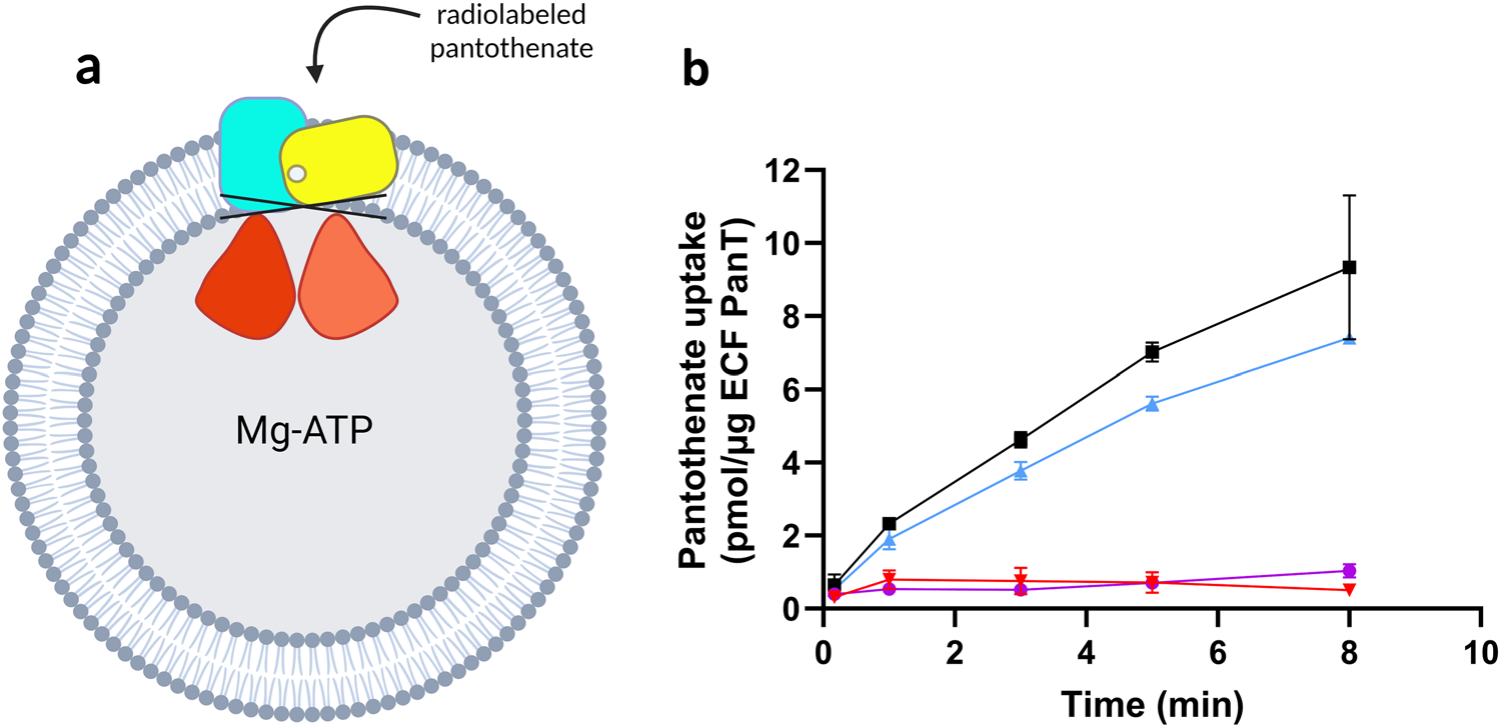
Effects of compounds **1** on the pantothenate transport-activity of ECF transporter ECF-PanT. **a** Schematic representation of uptake assay. **b** Pantothenate uptake by ECF PanT in proteoliposomes filled with 5 mM of MgATP (black), 5 mM of MgADP (purple), 5 mM MgATP plus 5 mM of compound **1** (red), and a final DMSO concentrations of 5% (v/v). The blue curve represents proteoliposomes filled with 5 mM MgATP and no DMSO. The error bars represent the standard deviation of three independent measurements, except for the data in panel B, where they represent the spread of the data from two independent measurements.

Encouraged by these results, we aimed to reinforce the idea that our chemical class might act also at the interface of ECF-PanT. We performed transport-activity assays similar to what we had done for ECF-Folt2 (Fig. 2), but now with ECF-PanT (Fig. 5), which is specific for panthothenate. This idea is based on the fact that the bacterium *L. delbrueckii* has eight different S-components that share the same ECF-module. ECF-PanT has the same ECF module as ECF-FolT2, but uses a different S-component to provide substrate specificity^39,40^. We purified ECF-PanT, reconstituted the protein complex in liposomes, and showed that compound **1** inhibited the uptake of pantothenate by this transporter, similar to what we had observed for inhibition of folate uptake by ECF-FolT2 (Fig. 2).

Given that both ECF complexes (FolT 2 and PanT) are inhibited by compound **1**, a reasonable assumption would be that both share the same ligand pocket. However, the structures of both complexes are not exactly the same, showing a slightly different orientation of the S-component^39,40^. Consequently, there are differences in the protein-protein interface between the S-component and the ECF module. Accordingly, pocket P9 varies between complexes with different S-components.

**Fig. 6:**
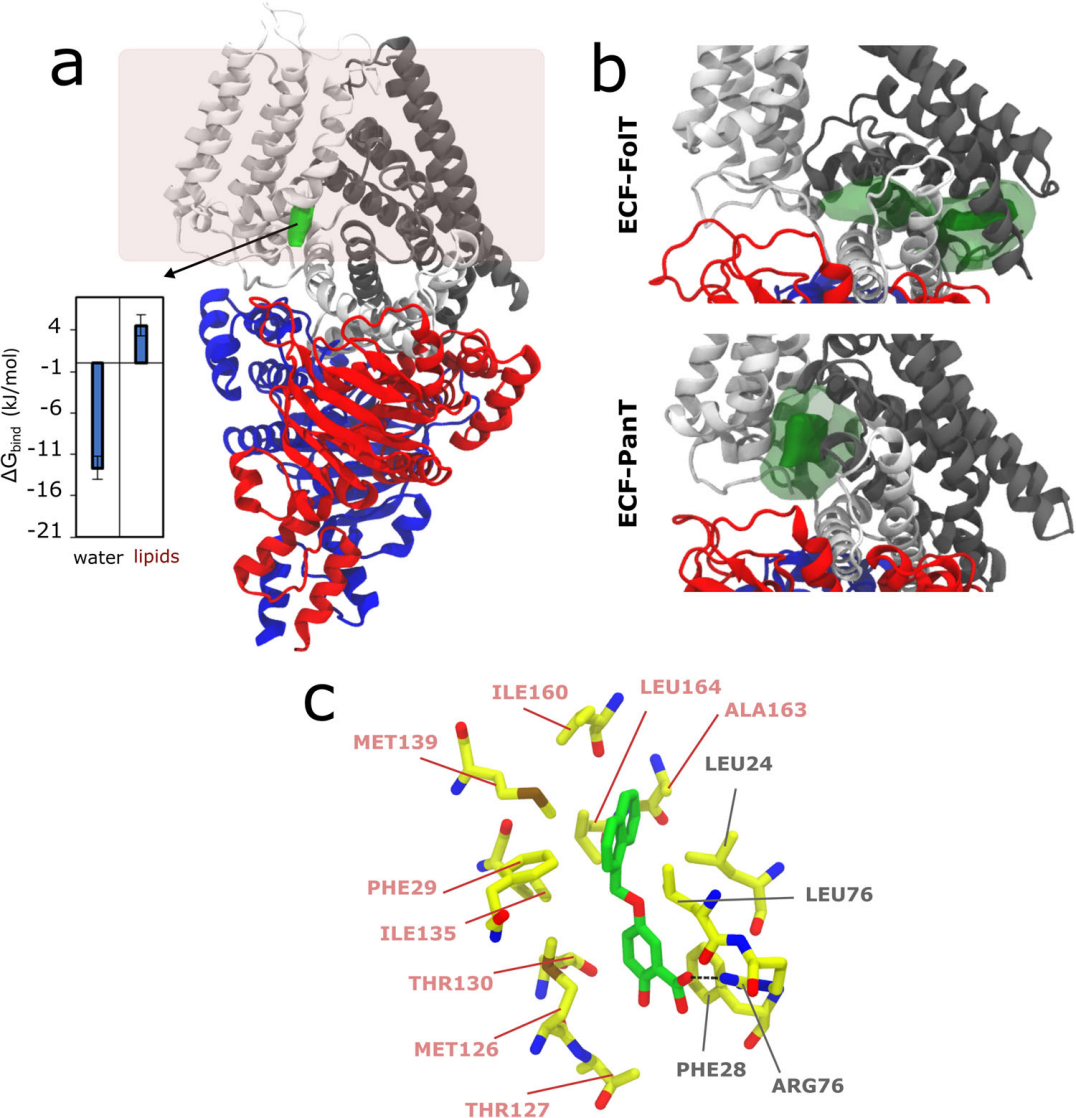
Unbiased CG molecular dynamics simulations of inhibitor binding to ECF transporter. **a** Crystal structure of ECF PanT (PDB ID 6ZG3)^40^, using the same color scheme and analysis as Fig. 3. Results indicated only one pocket for this ECF complex, which as P9 in ECF-FolT2 is at the interface between EcfT and the S-component. The bar plot shows calculated binding free energies (ΔG_bind_) of compound **1** to this pocket. The error bars represent the mean absolute error obtained by the block-averaging approach. **b** Compound **1**: pocket P9 density in ECF-FolT2 and the pocket of ECF-PanT simulations. The solid and transparent green isosurfaces correspond to a 100- and 10-fold higher density than in the bilayer. The proximity of the low occupancy isosurface indicates the proximity of the pockets. **c** Representative pose of compound **1** inside the pocket of ECF-PanT. The structures were backmapped from the CG to the atomistic resolution. Some residues from EcfT (salmon) and S-component (gray) are highlighted, including the hydrogen-bond network (black, dashed lines). Hydrogen atoms have been omitted for clarity.

To test the binding mode of hit **1** to ECF-PanT, we performed additional CG simulations. As expected by the closed state of the S-component, the results obtained show no binding to P11. Only one binding pocket was predicted in this case, located at the interface of the S-component and EcfT (Fig. 6a, with more details in Supplementary Fig. 15 and Supplementary Table 2). The ECF-PanT pocket and P9 in ECF-FolT are located in proximity of each other, which is more evident if we also consider the differences in the position of FolT and PanT S-components, which show a difference of 10 degrees in the orientation of transmembrane helices in relation to the ECF module^39,40^. Taking into consideration regions with lower ligand occupancy around these pockets (10 times less than the main pocket), the whole PPI region between S-component and EcfT may even be considered connected (Fig. 6b), which may explain how compound **1** can inhibit ECF bound to different S-components. This larger interface may be considered for fragment growing and optimization of compound **1** in the future.

Representative poses of compound **1** in P9 of ECF-FolT2 (Fig. 3b) and the pocket of ECF-PanT (Fig. 6c) show that polar/charged residues (such as Ser173 and Lys102 in the S-component of ECF-FolT and Arg76 of the S-component of ECF-PanT) can form a hydrogen bond with the salicylic acid moiety of compound, which reinforce the role of this substituent for the inhibitory activity. Contact analysis performed along the CG MD trajectories confirmed the importance of these interactions. In particular for ECF-FolT2, with compound **1** remaining 66% of the time bound to P9, with Ser172 and/or Lys102 of the S-component in close proximity to the carboxylic acid of the ligand. Similarly, compound **1** bound to ECF-PanT pocket also showed close contacts between Arg76 of the S-component and the same chemical group of the ligand, but less frequently, appearing in 30% of the time that the ligand resided in the pocket.

Next, to further corroborate the results obtained from MD analysis and experimentally validate the binding site of this chemical class, we conducted site-directed mutagenesis experiments. Therefore, Lys102 was mutated to either aspartic acid or glutamic acid. Although the mutated proteins behaved like wild-type proteins in production, purification, and reconstitution (Supplementary Notes 7, Supplementary Figs. 17 and 18), they showed a severe loss of transport activity preventing any conclusion on the binding site (Supplementary Figs. 19 and 20). Nonetheless, the loss of activity is consistent with the functional importance of the region targeted by the inhibiting compounds.

## Conclusions

In summary, we report the identification of the salicylic acid derivative of hit **1** as a new inhibitor of ECF transporters. Assessment of the inhibition of the ECF transporters supports the potential of this chemotype initially as a chemical probe and ultimately for the development of antibacterial agents that target the ECF transporters. Results from unbiased CG molecular dynamics simulations on *L. delbrueckii* ECF-FolT2 and ECF-PanT were in agreement with the design strategy of the newly synthesized molecules, showing that the ligand may bind at the interface involving the S-component and ECF module. The compound might therefore interfere with the protein-protein interface. Although our experimental data strongly indicate that the ECF transporter is the target and that the design hypothesis is correct, future work must include an experimental assessment of the binding pocket and inhibition mechanism of the compounds.

## Methods

### Biochemical assays

The target protein (ECF-FolT2 and -PanT from *Lactobacillus delbrueckii* subsp. *bulgaricus* or OpuA from *Lactococcus lactis*) was expressed, purified, and reconstituted in liposomes as described in more detail in the Supplementary Notes 3. To assess the compounds’ inhibitory impact on ECF-FolT2, ECF-PanT, and OpuA transport activity, we conducted a radiolabeled uptake assay, following established procedures described in Supplementary Notes 3. Proteoliposomes were prepared and loaded with 5 mM of Mg-ATP, 5 mM of Mg-ADP, or a combination of 5 mM of Mg-ATP and 5 mM of compound^29^. Additionally, the same concentration of the compound was introduced into the external solution. Proteoliposomes were present with 10% (v/v) DMSO inside the lumen of proteoliposomes and in the external solution.

### Synthesis and characterization of compounds

The compounds have been synthesized as described in the Supplementary Notes 4. All the compounds have been fully characterized by proton, carbon, and HRMS (Supplementary Figs. 6–13).

### Coarse-grained molecular dynamics simulations

All simulations were performed with the program package GROMACS (version 2018.8)^41^ and the Martini 3 Coarse-Grained (CG) force field^35^. The CG protein model was generated with Martinize2^42^ using the structures of the apo form of the ECF FolT2 (PDB code: 5JSZ)^29^ and ECF PanT (PDB ID 6ZG3)^40^ as initial references. A customized elastic network was used to keep the overall structure of ECF complexes. CG models of compounds **1,** **13**, and **14** were obtained according to the parametrization rules of Martini 3, as described elsewhere^34,35,43^. INSANE code^44^ was used to build the initial simulation boxes of ECF complexes embedded in a bacterial membrane model composed of 1-palmitoyl-2-oleoyl-sn-glycero-3-phosphoethanolamine (POPE), 1-palmitoyl-2-oleoyl-sn-glycero-3-phosphoglycerol (POPG) and cardiolipin (CL) in a ratio of 70:25:5. For each system (ECF FolT2 with compounds **1,** **13**, and **14** and ECF PanT with compound **1**), a total of 10 MD simulation replicas were performed, with each production simulation carried out for 30 µs. Therefore, in a total sampling of 300 µs was performed for each system. Analyses of the trajectories were performed with VMD^45^ and GROMACs tools (version 2018.8)^41^. Backmapping from CG representative snapshots to the atomistic resolution was performed with the backward code^46^. More technical details about the CG models, system setups, simulation settings, analysis, and backmapping are given in Supplementary Notes 5.

### Statistics and reproducibility

*For the radiolabel uptake assay:* For assessing the consistency and reliability of results, each uptake assay experiment was provided with technical replication three or two times for every time point. The measurement error was estimated as the standard deviation of three independent measurements or data points represented individually for two independent measurements.

*For the IC*_*50*_ *determination:* Each uptake assay experiment with different concentrations of the compound was provided with technical replication three times for every time point. The initial rates of transport activity were calculated from the slope of the folate uptake curve in the linear range (first 4 min) using simple linear regression in GraphPad Prism 10 with *R*^2^ ≥ 0.96. IC_50_ interpolation was provided with the dose-response equation in GraphPad Prism 10 with *R*^2^ ≥ 0.9.

*For the MD simulations:* In regard to the reproducibility, each system was simulated with 10 copies of standard MD. The error was estimated by a block-averaging approach. The total sampling for each system (300 µs) was divided into three equal blocks each containing 100 µs. The mean value for each block, mi (*i* = 1–3), was first calculated. The reported error bars are the mean absolute error of the means of each block (m1, m2, m3) in relation to the mean value obtained with the whole sampling.

### Reporting summary

Further information on research design is available in the Nature Portfolio Reporting Summary linked to this article.

### Supplementary information

Supplementary Information

Description of Additional Supplementary Files

Supplementary Data 1

Reporting Summary

## Data Availability

The source data behind the graphs can be found in Supplementary Data 1.

## Code availability

All software used for this study are available and the links are provided as follows. For virtual screening, it was used BioSolveIT https://www.biosolveit.de/ and DoGSiteScorer for druggability assessment. For MD simulations and analyses with GROMACS, the code is available at https://www.gromacs.org/. For visualization of the MD trajectories and analysis with VMD, https://www.ks.uiuc.edu/Research/vmd/. Martini CG Force-field parameters, codes, and procedures (e.g. tutorials) are publicly available at http://cgmartini.nl, https://mad.ibcp.fr/ and https://github.com/Martini-Force-Field-Initiative.
