# Author Correction: Identification of inhibitors targeting the energy-coupling factor (ECF) transporters

**DOI:** 10.1038/s42003-024-05770-0

**Published:** 2024-01-09

**Authors:** Eleonora Diamanti, Paulo C. T. Souza, Inda Setyawati, Spyridon Bousis, Leticia Monjas, Lotteke J.Y.M. Swier, Atanaz Shams, Aleksei Tsarenko, Weronika K. Stanek, Manuel Jäger, Siewert J. Marrink, Dirk J. Slotboom, Anna K. H. Hirsch

**Affiliations:** 1 Helmholtz Institute for Pharmaceutical Research (HIPS) − Helmholtz Centre for Infection Research (HZI), Campus Building E 8.1, D-66123 Saarbrücken, Germany; 2 Molecular Microbiology and Structural Biochemistry, UMR 5086 CNRS and University of Lyon, Lyon, France; 3 Laboratoire de Biologie et Modélisation de la Cellule (UMR 5239, Inserm, U1293) and Centre Blaise Pascal, École Normale Supérieure de Lyon, Université Claude Bernard Lyon 1 and CNRS, 46 Allée d’Italie, 69007 Lyon, France; 4 Biomolecular Sciences and Biotechnology Institute, University of Groningen, Nijenborgh 4, 9747AG Groningen, The Netherlands; 5 Department of Biochemistry, Bogor Agricultural University, Dramaga, 16680 Bogor, Indonesia; 6 Department of Pharmacy, Saarland University, Campus Building E8.1, 66123 Saarbrücken, Germany; 7 Stratingh Institute for Chemistry, University of Groningen, Nijenborgh 7, NL-9747 AG Groningen, The Netherlands

**Keywords:** Molecular modelling, Bacterial infection

Correction to: *Communications Biology* (2023) **6**:1182; 10.1038/s42003-023-05555-x

In this article the author name Leticia Monjas was incorrectly written as Leticia M. Gómez. The original article has been corrected.

