# Supplementary material for: Identification of inhibitors targeting the energy-coupling factor (ECF) transporters
Source: Commun Biol. 2023 Nov 20;6:1182. doi: 10.1038/s42003-023-05555-x (PMC10662466; doi:10.1038/s42003-023-05555-x)
Supplement: Supplementary file 1 — Supplementary Information [file 42003_2023_5555_MOESM1_ESM.pdf]

Supporting Information

Identification of inhibitors targeting the energy-coupling factor (ECF) transporters

Eleonora Diamanti,[‡a] Paulo C. T. Souza,[‡b,c] Inda Setyawati,[d,e] Spyridon Bousis,[a,f,g] Leticia M. Gómez,[g] Lotteke J.Y.M. Swier,[d] Atanaz Shams,[a] Aleksei Tsarenko,[d] Weronika K. Stanek,[d] Manuel Jäger,[g] Siewert J. Marrink,[d] Dirk J. Slotboom[d] and Anna K. H. Hirsch*[a,f,g]

[a] Department for Drug Design and Optimization, Helmholtz Institute for Pharmaceutical Research (HIPS) − Helmholtz Centre for Infection Research (HZI), Campus Building E 8.1, D-66123, Saarbrücken, Germany.
[b] Molecular Microbiology and Structural Biochemistry, UMR 5086 CNRS and University of Lyon, Lyon, France.
[c] Laboratoire de Biologie et Modélisation de la Cellule (UMR 5239, Inserm, U1293) and Centre Blaise Pascal, École Normale Supérieure de Lyon, Université Claude Bernard Lyon 1 and CNRS, 46 Allée d'Italie, 69007, Lyon, France.
[d] Biomolecular Sciences and Biotechnology Institute University of Groningen Nijenborgh 4, 9747AG Groningen, The Netherlands.
[e] Department of Biochemistry, Bogor Agricultural University, Dramaga, 16680 Bogor, Indonesia.
[f] Department of Pharmacy, Saarland University, Campus Building E8.1, 66123 Saarbrücken, Germany.
[g] Stratingh Institute for Chemistry, University of Groningen, Nijenborgh 7, NL-9747 AG Groningen, the Netherlands.

‡These authors contributed equally to this work.

*Corresponding author
Prof. A. K. H. Hirsch
Helmholtz Institute for Pharmaceutical Research Saarland (HIPS)-Helmholtz Centre for Infection Research (HZI)
Department of Drug Design and Optimization
Campus Building E8.1, 66123 Saarbrüucken, Germany
e-mail: anna.hirsch@helmholtz-hzi.de

Table of Contents

Supplementary Notes 1 - Prediction of druggable pockets 3

Supplementary Notes 2 - Structure-based Virtual Screening (SBVS) 4

Supplementary Notes 3 - Biochemical assays 7

Supplementary Notes 4 - Synthesis and characterization of compounds 1, 13 and 14. 10

5.1 General information 10

5.2 Synthetic schemes 11

5.3 General procedure 11

5.4 ^{1}H-, ^{13}C-NMR spectra, HR-MS and LC-MS analysis of compounds 1, 2, 13, 14. 13

Supplementary Notes 5 - Coarse-grained molecular dynamics simulations 23

Supplementary Notes 6 - Docking Studies in SeeSAR 27

Supplementary Note 7 - Site-directed mutagenesis 29

Supplementary References 31

Supplementary Notes 1 - Prediction of druggable pockets

The DoGSiteScorer server was used to find pockets on the surface of ECF-FolT2 and predict their druggability[1]. DoGSiteScorer was customized to work on the pocket level with the druggability estimation for pockets option switched on. Thirty-six pockets were found, of which eleven were considered to be druggable[2].

The eleven druggable pockets identified are scattered over the whole surface of the ECF-FolT2 (Supplementary Figure 1)[3].

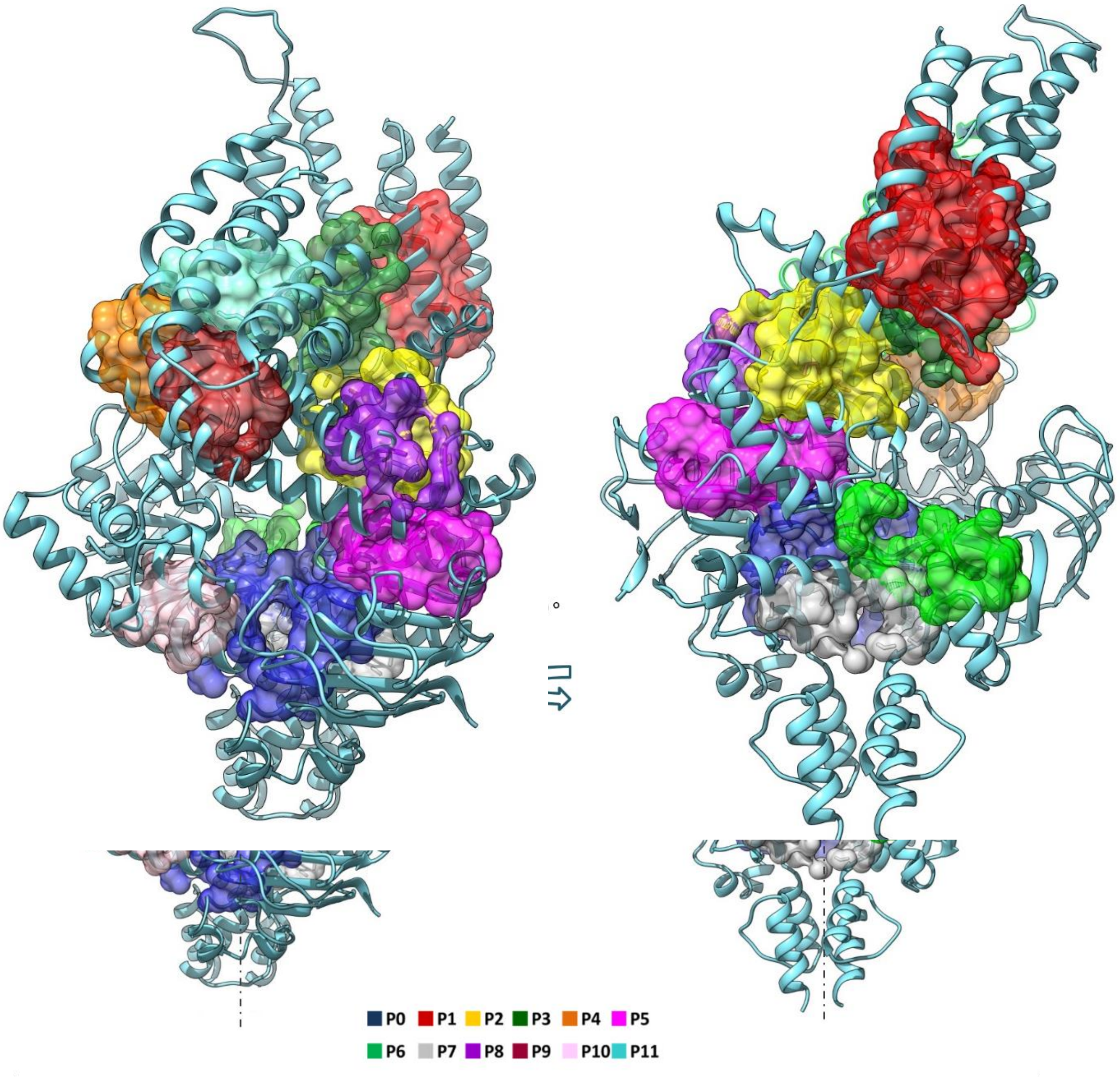

Supplementary Figure 1 ECF-FolT transporter (PDB code 5JSZ) is represented in cartoon and colored in cyan. Side view of ECF-FolT along with the 12 identified pockets: P0, P1, P2, P3, P4, P5, P6, P7, P8, P9, P10 and P11 colored in medium blue, red, yellow, forest green, orange, magenta, green, grey, purple, firebrick, pink and aquamarine respectively. Images were visualized in CHIMERA software. The color coding of the pockets and the ECF-FolT transporter is maintained throughout the manuscript for figures showing the ECF transporter in ribbon.

The pocket P2 (yellow pocket, in Supplementary Figure 1) has been chosen among the twelve pockets as the most interesting and promising one. It is located in the EcfT domain and lined by residues from coupling helix 1 (CH1), the *N*-terminal part of coupling helix 2 (CH2) of EcfT and the loop L7 connecting coupling helix 3 (CH_3) of EcfT with the final transmembrane helix of this protein.

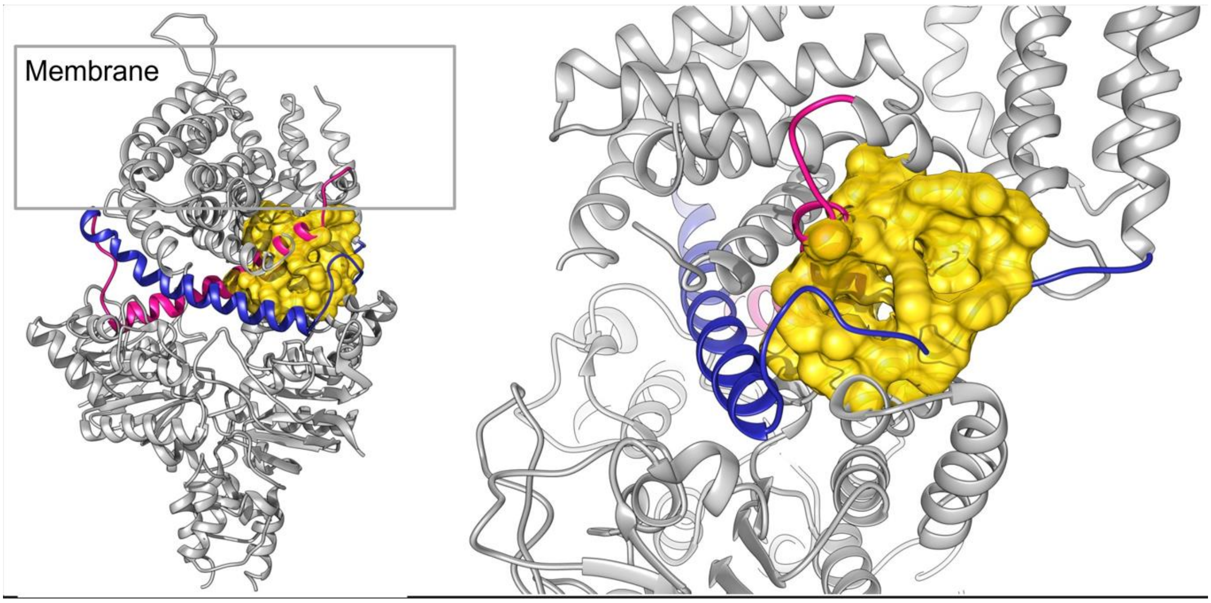

Supplementary Figure 2: Cartoon representation of ECF FolT2 in the *apo* state (PDB ID 5JSZ) along with the two coupling helices CH2 and CH3 in pink and blue, respectively, and the P2 pocket depicted as yellow surface. The approximate location of the membrane is indicated by the rectangle.

Supplementary Notes 2 - Structure-based Virtual Screening (SBVS)

Virtual Screening Compounds. The compounds used for the virtual screening were from the Express Collection of Princeton BioMolecular Research (http://www.princetonbio.com/, downloaded in May 2015). Initially, the library was filtered in MONA according to the following criteria: molecular weight 250–500 g/mol, aromatic fused tricyclic (or bigger) compounds and esters were removed [4,5].

Protein Preparation. The crystal structure of ECF FolT2 in the *apo* state (PDB ID 5JSZ) was obtained from the protein data bank (PDB) and prepared for structure-based virtual screening[6]. First, the receptor was defined by manual selection including the following amino acid residues: F97–P118 and E140–S142 from EcfA; L69, V70, V73 and I74 from FolT2; P132–T169 and L218–L239 from EcfT.

Virtual Screening. The library was screened using the KNIME Analytics Platform[7] with the help of the suggested workflow and nodes (SeeSAR, Generate 3D Coordinates, LeadIT) from BioSolveIT[8].

Next, the 3D-coodinates were generated by the 3D generator, and the library was docked by using LeadIT (version 2.1.8) [9].The default settings were used for docking, and ten poses of each molecule were generated; promiscuous and toxic

compounds were filtered out using the PAINS-filter [10] and Eli Lilly-rules [11, 12].The resulting poses were scored with HYDE in SeeSAR (version 3.2) [13] and ranked according to the estimated binding affinity. Poses with red flagged torsions as well as compounds giving only one pose were removed. From the first 1000 entries, the best 100 compounds were visually inspected in SeeSAR, and the ones with at least two similar poses, which belong to different clusters, were ordered from Princeton BioMolecular Research, Inc. for biochemical evaluation.

Characterization of compounds purchased. The compounds were purchased in 10 mg batches and directly dissolved in DMSO without further purification, as to obtain 100 mM solutions. Their solubility in the assay buffer was tested prior to performing the uptake assay as to reach 5 mM as the highest concentration tested in 50 mM KPi pH 7.5 with a final DMSO concentration of 5% (v/v). Most of the compounds precipitated under these conditions, except for compound **1**, **3** and **11,** which were subsequently used in the uptake assay (Supplementary Figure 4A). Upon increasing the DMSO concentration to a final concentration of 10% (v/v) in 50 mM KPi pH 7.5, compounds **2**, **5** and **8** showed to be soluble as well, and were therefore used in an uptake assay performed under these conditions (Supplementary Figure 4B). The most active compounds (**1**–**12**) were re-ordered in higher amount (typically 100 mg) from Princeton Biomolecular Research and analyzed by NMR and MS prior to IC_{50} determination.

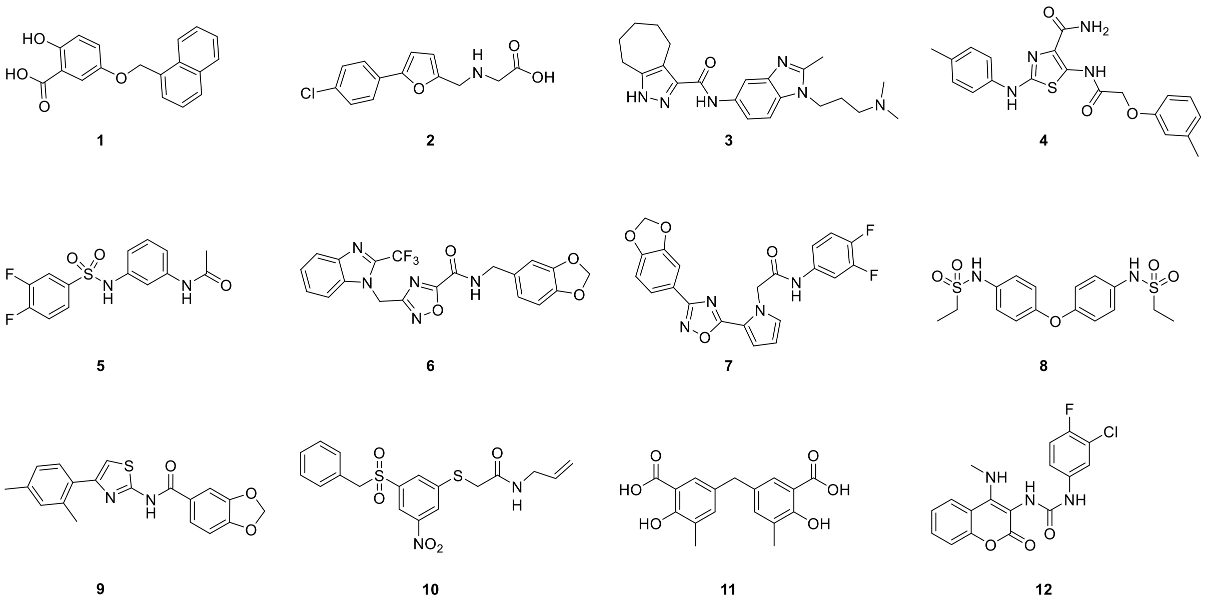

Supplementary Figure 3. Twelve compounds selected from the SBVS.

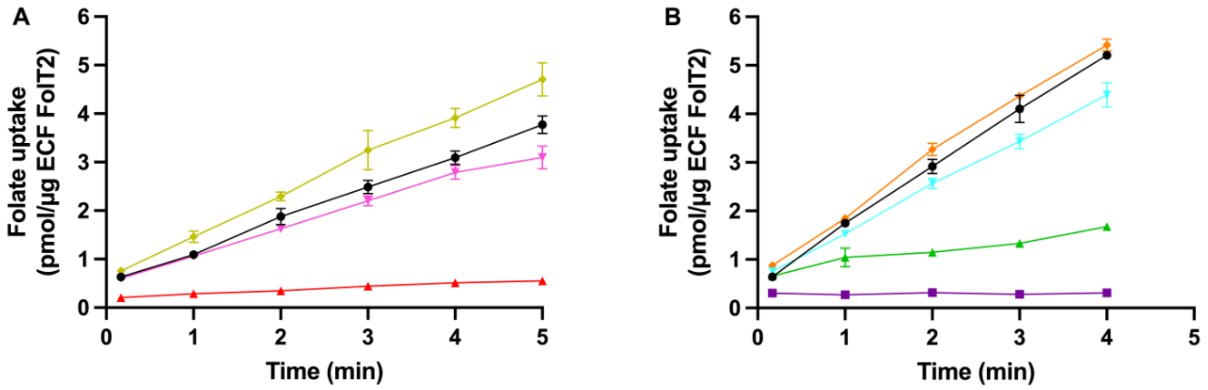

Supplementary Figure 4. Effects of the compounds on folate uptake by ECF-FolT2. (A) Folate uptake by ECF-FolT2 in proteoliposomes filled with 5 mM of MgATP, 5 mM of compound **1** (red), **3** (magenta), **11** (yellow), or no compound (black) and a final DMSO concentration of 5% (v/v). **(B)** Same as in Panel A, with proteoliposomes filled with 5 mM of MgATP, 5 mM of compound **2** (green), **5** (light blue), **8** (orange), no compound (black) or 5 mM of MgADP (purple) and a final DMSO concentration of 10% (v/v). The errors in the graphs represent the standard deviation from three experiments.

Supplementary Notes 3 - Biochemical assays

Expression, purification and reconstitution of ECF-FolT2 and -PanT in proteoliposomes.

The expression, purification and reconstitution of ECF-FolT2 and -PanT in proteoliposomes was performed as described previously[6]. Briefly, ECF-FolT2/-PanT was produced in *Escherichia coli* MC1061, which had been transformed with p2BAD vectors. The genes encoding EcfA, EcfA' and EcfT of *L. delbrueckii* subsp. *Bulgaricus* are annotated as *cbiO* (both for LDB_RS01805, *ecfA* and LDB_RS01810 *ecfA'*) and *cbiQ* (LDB_RS01815, *ecfT*). *folT2* (LDB_RS07030) or *panT* (LDB_RS01970) was cloned downstream of the second promoter. The expression of ECF-FolT2/-PanT was conducted in a 5-liter flask containing 2 liters of yeast tryptone medium (8 g/l Bacto tryptone, 5 g/l Bacto yeast extract, and 2.5 g/l NaCl), 2.5 mM potassium phosphate (KPi) at pH 7.0, 0.5% glycerol, and 100 mg/ml ampicillin. E. coli MC1061 cells harboring p2BAD His10-ECF-FolT2-STREPII or p2BAD His10-ECF-PanT-STREPII were cultured at 37°C and 200 rpm until reaching an OD_{600} of 0.8. At this point, the temperature was reduced to 25°C. After a 20-minute cooling period, we induced expression by adding 1.0 x 10^-2% arabinose. Following 3 hours of expression, the cells were harvested through centrifugation (15 minutes at 7,446g, 4°C), washed with 50 mM KPi, pH 7.5 buffer, and resuspended in 50 mM KPi, pH 7.5, 10% glycerol. Membrane vesicles were either prepared immediately or, alternatively, the resuspended cells were flash-frozen in liquid nitrogen and stored at -80°C. Purification was also done as previously described[6]. Briefly, membrane vesicles were solubilized in 50 mM KPi, pH 7.5, 300 mM NaCl, 15 mM imidazole, 1% (w/v) *n*-dodecyl-β-D-maltopyranoside (DDM, Anatrace) at 4 °C for 1 h, while gently nutating. Unsoluble material was removed by centrifugation at 4 °C for 20 min and 442,907*g*,. Solubilized material was incubated at 4 °C for 1 h with 0.5 mL Ni^{2+}-sepharose resin (equilibrated with 50 mM KPi, pH 7.5, 300 mM NaCl, 50 mM imidazole, 0.05% (w/v) DDM) while nutating. The column material was washed with 15 mL of the same buffer. The ECF-FolT2 complex was eluted in 50 mM KPi, pH 7.5, 300 mM NaCl, 500 mM imidazole, 0.05% (w/v) DDM, and 1 mM of EDTA was added to the eluate. The protein was further purified by size-exclusion chromatography using a Superdex 200 10/300 gel filtration column (GE Healthcare), equilibrated with buffer 50 mM KPi, pH 7.5, 150 mM NaCl, 0.05% (w/v) DDM. Purified ECF-FolT2/-PanT was reconstituted in proteoliposomes as described previously[14], using a protein to lipid (w/w) ratio of 1:250 and 1:1000 (for ECF-FolT2 and ECF-PanT respectively) and using liposomes composed of *E. coli* polar lipids and egg phosphatidylcholine (3:1 (w/w) ratio).

Expression, purification and reconstitution of OpuA in proteoliposomes, and radiolabeled uptake assay

The expression, purification and reconstitution of OpuA in proteoliposomes was performed as described elsewhere, using a protein to lipid reconstitution ratio of 1:100 (w/w)[14,15].

Radiolabelled folate and pantothenate transport assay

For determining the inhibitory effect of the compounds on the transport activity of ECF-FolT2/-PanT, a radiolabeled uptake assay was performed as described previously[6]. The proteoliposomes were loaded with 5 mM of Na_2-ATP plus 5 mM $MgSO_4$, 5 mM Na_2-ADP plus 5 mM $MgSO_4$ or 5 mM of Mg-ATP plus 5 mM or 0.25 mM compound. To incorporate these compounds into the proteoliposomes, a total of three cycles of flash-freezing in liquid nitrogen and rapid thawing were performed. Subsequently, the proteoliposomes were extruded through a 400-nm pore size polycarbonate filter (Avestin) for 11 passages. This step was likely performed to ensure uniformity and size consistency of the liposomes. To remove external nucleotides, the proteoliposomes were diluted to reach a final volume of 6 ml. After this, centrifugation was carried out for 30 minutes at 285,775g and 4°C. Following centrifugation, the proteoliposomes were resuspended in 50 mM KPi, pH 7.5 to achieve a final concentration of 0.5–1 µg/µl ECF-FolT2/PanT. The compounds were also added in the external solution with the same

concentration. Both inside the lumen of proteoliposomes and in the external solution 5 or 10% (v/v) of DMSO was present. The transport was initiated by adding 1 µg of ECF-FolT2 or 0.25 µg ECF-PanT, reconstituted in proteoliposomes in reaction mixture containing 50 mM KPi, pH 7.5, 95 nM of non-radiolabeled substrate and 5 nM of [3,5,7,9-3H] radiolabeled folate or 5 nM of D-[2,3-^{3}H]pantothenic acid. At specified time points, 2 ml of ice-cold stop buffer (50 mM KPi, pH 7.5) was added, and the reaction was quickly filtered over a BA-85 nitrocellulose filter. After washing the filter with an additional 2 ml of stop buffer, the filter was dried for 1 hour. Subsequently, the filter was dissolved in 5 ml of Filter Count scintillation liquid (PerkinElmer), and the radioactivity levels were determined using a PerkinElmer Tri-Carb 2800 TR isotope counter.

Radiolabeled glycine betaine transport assay

The radiolabeled uptake assay was performed as described previously, using proteoliposomes loaded with 5 mM of Na_2-ATP plus 5 mM $MgSO_4$ instead of the ATP-regenerating system[16]. Additionally, the proteoliposomes were loaded with 5% (v/v) of DMSO, 5 mM of compound **1** in DMSO with a final DMSO concentration of 5% (v/v), or an equivalent volume of buffer. Encapsulation of the chemicals was achieved by performing five freeze-thaw cycles, followed by two washing steps with 50 mM KPi, pH 7.0, after which the proteoliposomes were resuspended to a final OpuA concentration of about 80 mg/mL. For the uptake assay, the proteoliposomes were diluted to a final OpuA concentration of 5 mg/mL in 47 mM KPi, pH 7.0, 500 mM KCl, 4.8 µM of [^{14}C]-glycine betaine, 14.9 µM of non-radiolabeled glycine betaine, and the reaction was followed for five minutes.

IC_{50} determination

For determination of the apparent IC_{50} values of compounds **1** and **2**, the ECF-FolT2 proteoliposomes and the uptake assays were prepared and performed as described above. However, the proteoliposomes were loaded with concentrations of the compounds ranging from $2.0*10^{-2}$ to 7.0 mM in the presence of 10% (v/v) of DMSO. The compounds were also added in the external solution for compound **1**, but not for compound **2**. The activity in the presence of series of concentrations of compounds was compared to an uninhibited control. The IC_{50} estimation values were fitted with Dose Response equation using GraphPad Prism 10.

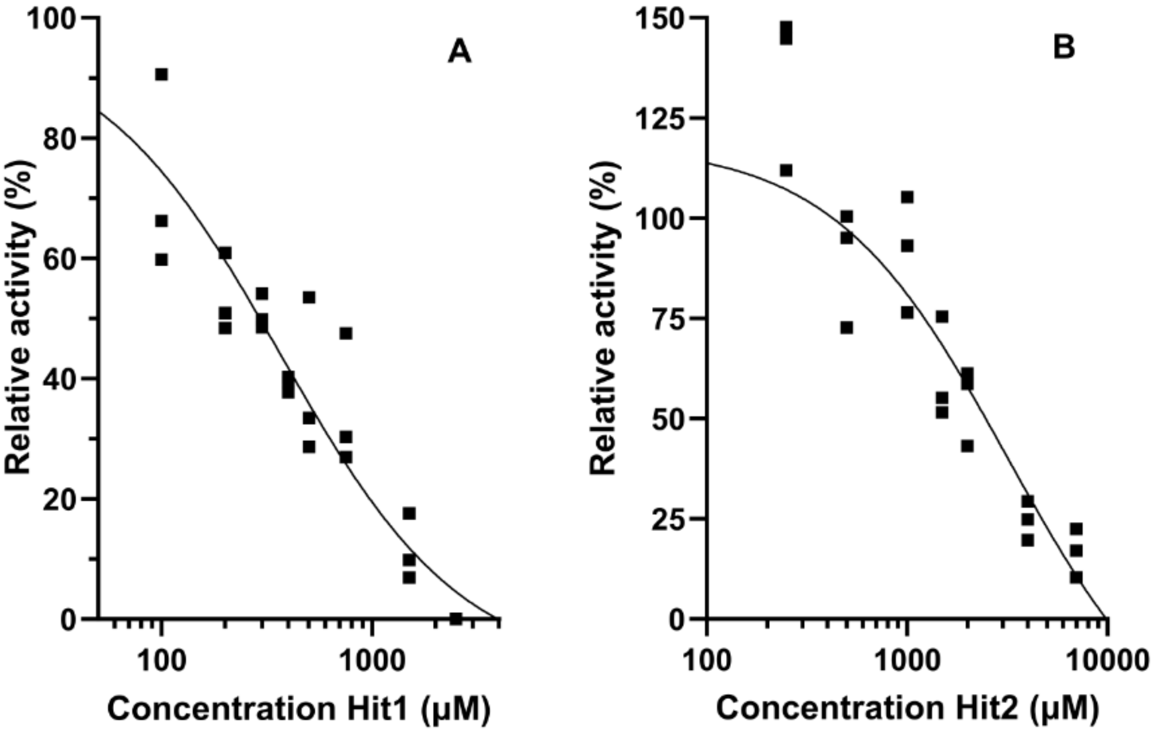

Supplementary Figure 5. Determination of apparent IC_{50} values of compounds 1 (A) and 2 (B). The transport activity (initial rates) is shown at different inhibitor concentrations, as percentage of the activity without inhibitor. The initial rates were calculated from the slope of the folate uptake curve in the linear range (first 4 min). The errors in the graphs represent the standard deviation from three experiments. IC_{50} value of compound **1** = 282 ± 108 µM; **2** = 1.2 mM[19].

Supplementary Notes 4 - Synthesis and characterization of compounds 1, 13 and 14.

5.1 General information

Chemicals, Materials and Methods

NMR experiments were run on a Bruker Ultrashield plus 500 (500 MHz) spectrometer. Spectra were acquired at 300 K, using deuterated dimethylsulfoxide (DMSO-d_6) as solvent. Chemical shifts for ^{1}H and ^{13}C spectra were recorded in parts per million (ppm) using the residual non-deuterated solvent as the internal standard (for DMSO-d_6: 2.50 ppm, ^{1}H; 39.52 ppm, ^{13}C). Coupling constants (J) are given in Hertz (Hz). Data are reported as follows: chemical shift, multiplicity (s = singlet, d =doublet, t = triplet, m = multiplet, br = broad and combinations of these) coupling constants and integration. Flash chromatography was performed using the automated flash chromatography system CombiFlash Rf+ (Teledyne Isco, Lincoln, NE, USA) equipped with RediSepRf silica columns (Axel Semrau, Sprockhövel Germany). TLC was performed with aluminum-backed silica TLC plates (Macherey-Nagel MN ALUGRAM Sheets SIL G/UV 254 20 x 20cm 818133) with a suitable solvent system and was visualized using UV fluorescence (254 & 366 nm). All reactions were carried out in oven-dried glassware under an atmosphere of argon. Anhydrous DMF was purchased from Aldrich and used directly.

Mass spectrometry was performed on a SpectraSystems-MSQ LCMS system (Thermo Fisher, Dreieich, Germany) using a Hypersil Gold column, 150 x 3 mm, 5 µm. At a flow rate of 700 µL/min, the gradient of H_2O (0.1% FA) and ACN (0.1% FA) starting from 30% ACN and then increased to 95% over 12 min. The mass spectrum was measured in positive and negative mode in a range from 100–600 m/z. The UV spectrum was recorded at 254 nm. High-resolution mass spectra (HR-MS) were recorded with a ThermoScientific system where a Dionex Ultimate 3000 RSLC was coupled to a Q Exactive Focus mass spectrometer with an electrospray ion (ESI) source. An Acquity UPLC® BEH C8, 150 x 2.1 mm, 1.7 µm column equipped with a VanGuard Pre-Column BEH C8, 5 x 2.1 mm, 1.7 µm (Waters, Germany) was used for separation. At a flow rate of 250 µL/min, the gradient of (A) H_2O + 0.1% FA and (B) ACN + 0.1% FA was held at 10% B for 1 min and then increased to 95% B over 4 min. It was held there for 1.2 min before the gradient was decreased to 10% B over 0.3 min where it was held for 1 min. The mass spectrum was measured in positive mode in a range from 120–1000 m/z. UV spectrum was recorded at 254 nm. The compounds which did not give a good ionization at the Q Exactive, have been measured on a Dionex Ultimate 3000 RSLC system using a BEH C18, 100 x 2.1 mm, 1.7 µm dp column (Waters, Germany). Separation of 1 µl sample was achieved by a linear gradient from (A) H_2O + 0.1% FA to (B) ACN + 0.1% FA at a flow rate of 600 µL/min and 45 °C. The gradient was initiated by a 0.5 min isocratic step at 5% B, followed by an increase to 95% B in 18 min to end up with a 2 min step at 95% B before re-equilibration under the initial conditions. UV spectra were recorded by a DAD in the range from 200 to 600 nm. The LC flow was split to 75 µL/min before entering the maXis 4G hr-ToF mass spectrometer (Bruker Daltonics, Germany) using the Apollo ESI source. Mass spectra were acquired in centroid mode ranging from 150–2500 m/z at a 2 Hz scan rate.

Abbreviations dimethylformamide (DMF), dimethylsulfoxide (DMSO), ethyl acetate (EtOAc), hydrochloric acid (HCl), potassium carbonate (K_2CO_3), sodium sulfate (Na_2SO_4), sodium hydride 60% dispersion in mineral oil (NaH 60%). Other abbreviations used are: aqueous (aq.), hours (h), minutes (min), room temperature (rt), on (overnight), saturated (sat.).

5.2 Synthetic schemes

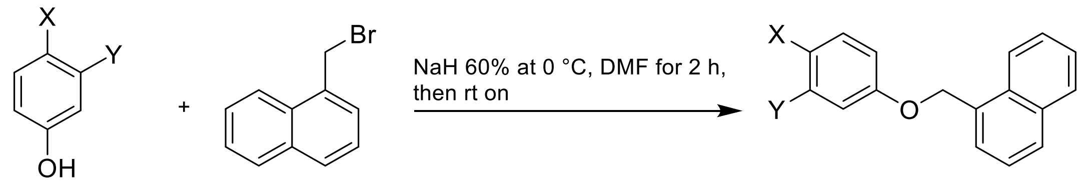

X = OH; Y = COOH
X = OH; Y = H

1: X = OH; Y = COOH (59%)
13: X = OH; Y = H (49%)
14: X = NHBoc; Y = COOH (45%)

Scheme S1: General procedure for the synthesis of compounds **1**

5.3 General procedure

General procedure 1 (GP1): ether synthesis

To a stirred solution of 5-hydroxy-2-substituted benzoic acid (1 eq) in DMF (0.3 M), was added at 0 °C portionwise NaH (60%, 2.5 eq) previously washed with diethyl ether (3x5 mL). The reaction mixture was stirred at rt for 2 h, and then 1-(bromomethyl)naphthalene (1 eq) was slowly added at 0 °C. The reaction mixture was stirred at rt on until TLC and LC-MS analysis showed the complete consumption of the starting materials. The resulting suspension was quenched at 0 °C with HCl (6 N) and extracted with EtOAc (3x10 mL). The combined organic layers were dried over Na_2SO_4, filtered, concentrated *in vacuo* and purified by flash chromatography.

Regioselective mono-O-alkylation: the one-step preparation is possible due to the difference in pK_a of the hydroxyl groups in positions 2 and 5. The selective mono-alkylation at position 5 has been confirmed by complete characterization of compound **1** by HMBC and NOESY experiments [20].

2-Hydroxy-5-((naphthalen-1-yloxy)methyl)benzoic acid (1)

According to GP1, using 2,5-dihydroxybenzoic acid (0.200 g, 1.3 mmol), NaH (0.130 g, 3.24 mmol) and naphthylbromide (0.287 g, 1.3 mmol) in DMF (4.3 mL) to give, after purification by column chromatography (hexane/EtOAc 1:9) **1** as white powder (0.225 g, 59%). **^{1}H NMR (500 MHz, DMSO-*d*$_6$)** δ 8.10 (d, *J* = 8.1, 1H), 7.99 – 7.95 (m, 1H), 7.93 (d, *J* = 8.2, 1H), 7.65 (d, *J* = 6.5, 1H), 7.61 – 7.54 (m, 2H), 7.51 (dd, *J* = 8.2, 7.0, 1H), 7.45 (d, *J* = 3.2, 1H), 7.28 (dd, *J* = 9.0, 3.2, 1H), 6.92 (d, *J* = 9.0, 1H), 5.51 (s, 2H). **^{13}C NMR (126 MHz DMSO-*d*$_6$)** 171.6, 155.6, 150.7, 133.3, 132.5, 131.2, 128.7, 128.5, 126.7, 126.5, 125.9, 125.4, 124.3, 123.98, 118.1, 114.1, 112.7, 68.5. **HR-MS (ESI)** calcd for $C_{18}H_{13}O_4$ [*M*–H]$^-$: 293.0892, found: 293.0818.

4-(Naphthalen-1-ylmethoxy)phenol (13)

To a solution of hydroquinone (0.100 g, 0.90 mmol) and K_2CO_3 (0.063 g, 0.453 mmol) in acetone (1.8 mL), naphthylbromide (0.100 g, 0.453 mmol) was added dropwise. The solution mixture was heated under reflux overnight. Once cooled, H_2O was added, and the reaction mixture was extracted with EtOAc (3x10 mL). The combined organic layers were dried over Na_2SO_4, filtered, concentrated *in vacuo* and purified by flash chromatography (hexane/EtOAc 6:4) to give **13** as a white powder (0.055 g, 49%). **^{1}H NMR (500 MHz, DMSO-*d*$_6$)** δ 8.95 (s, 1H), 8.09 (d, *J* = 8.1, 1H), 7.97 (d, *J* = 8.0, 1H), 7.92 (d, *J* = 8.2, 1H), 7.63 (d, *J* = 6.9, 1H), 7.59 – 7.53 (m, *J* = 21.2, 2H), 7.50 (t, *J* = 7.6, 1H), 6.90 (dd, *J* = 9.7, 2.9, 2H), 6.69 (d, *J* = 8.8, 2H), 5.42 (s, 2H). **^{13}C NMR (126 MHz DMSO-*d*$_6$)** 151.4, 151.2, 133.2, 132.9, 131.1, 128.5, 128.4, 126.5, 126.3, 125.9, 125.3, 123.9, 115.8, 115.7, 68.4. **HR-MS (ESI)** calcd for $C_{17}H_{15}O_2$ [*M*+H]$^+$: 251.1066, found: 251.1055.

2-((*tert*-Butoxycarbonyl)amino)-5-((naphthalen-1-yloxy)methyl)benzoic acid (14)

According to GP1, using 2-*tert*-butoxycarbonylamino-5-hydroxy-benzoic acid (0.500 g, 1.97 mmol), NaH (0.197 g, 4.92 mmol) and naphthylbromide (0.436 g, 1.97 mmol) in DMF (6.5 mL) to give, after purification by column chromatography (Hexane/EtOAC 2:8), **14** as light-yellow powder (0.35 g, 45%). **^{1}H NMR (500 MHz, DMSO-*d*$_6$)** δ 10.26 (s, 1H), 8.18 (d, *J* = 9.2, 1H), 8.10 (d, *J* = 8.1, 1H), 8.00 – 7.96 (m, 1H), 7.93 (d, *J* = 8.2, 1H), 7.67 (d, *J* = 6.8, 1H), 7.61 (d, *J* = 3.1, 1H), 7.57 (ddd, *J* = 7.9, 7.4, 1.3, 1H), 7.51 (dd, *J* = 8.0, 7.2, 1H), 7.35 (dd, *J* = 9.2, 3.1, 1H), 5.55 (s, 1H), 1.47 (s, 9H).**^{13}C NMR (126 MHz DMSO-*d*$_6$)** 169.2, 152.5, 152.1, 135.0, 133.2, 132.3, 131.1, 128.7, 128.4, 126.7, 126.4, 126.0, 125.3, 123.9, 121.3, 119.9, 116.9, 116.1, 79.8, 68.2, 28.0. **HR-MS (ESI)** calcd for $C_{23}H_{22}NO_5$ [*M*–H]$^-$: 392.1576, found: 392.1503.

5.4 ^{1}H-, ^{13}C-NMR spectra, HR-MS and LC-MS analysis of compounds 1, 2, 13, 14.

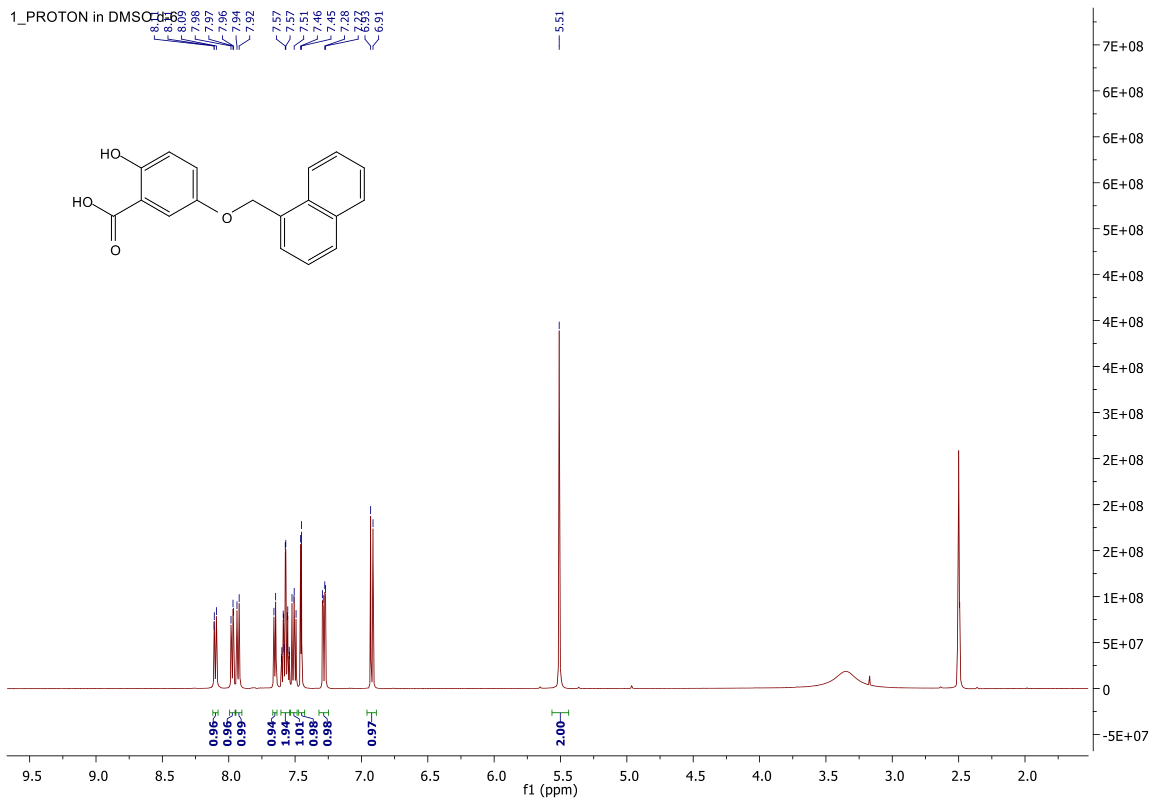

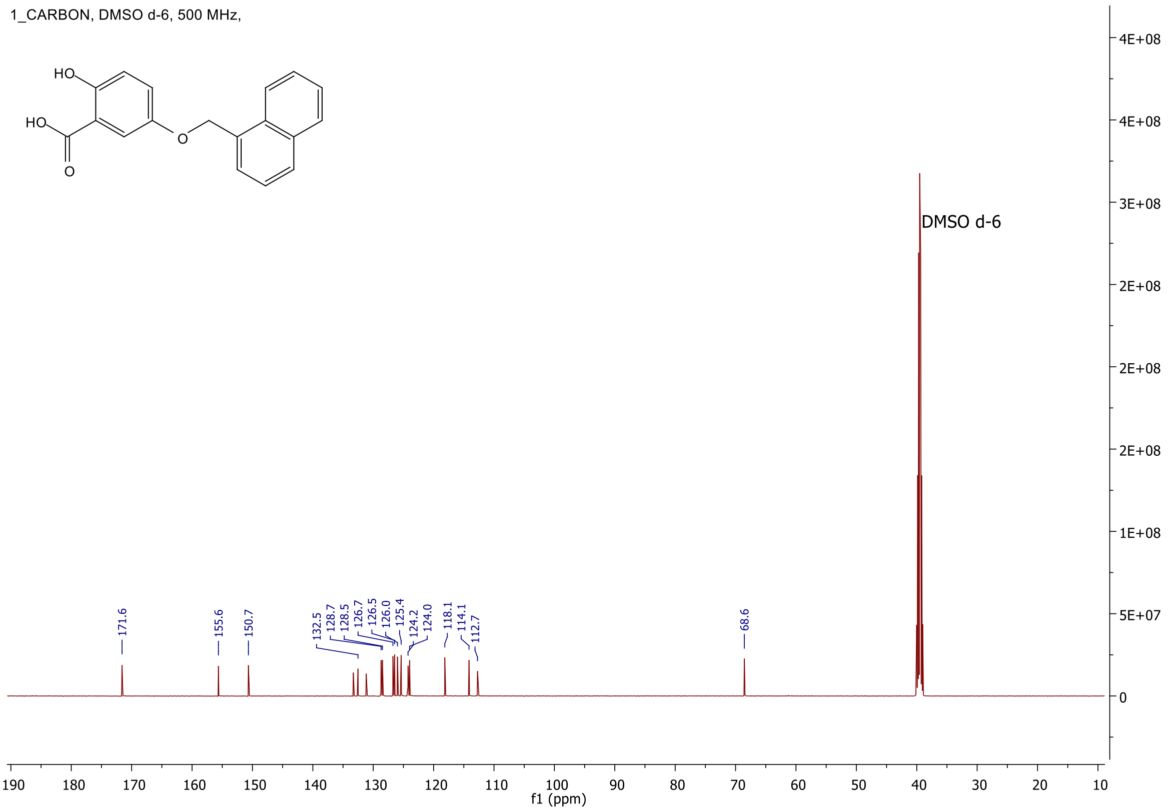

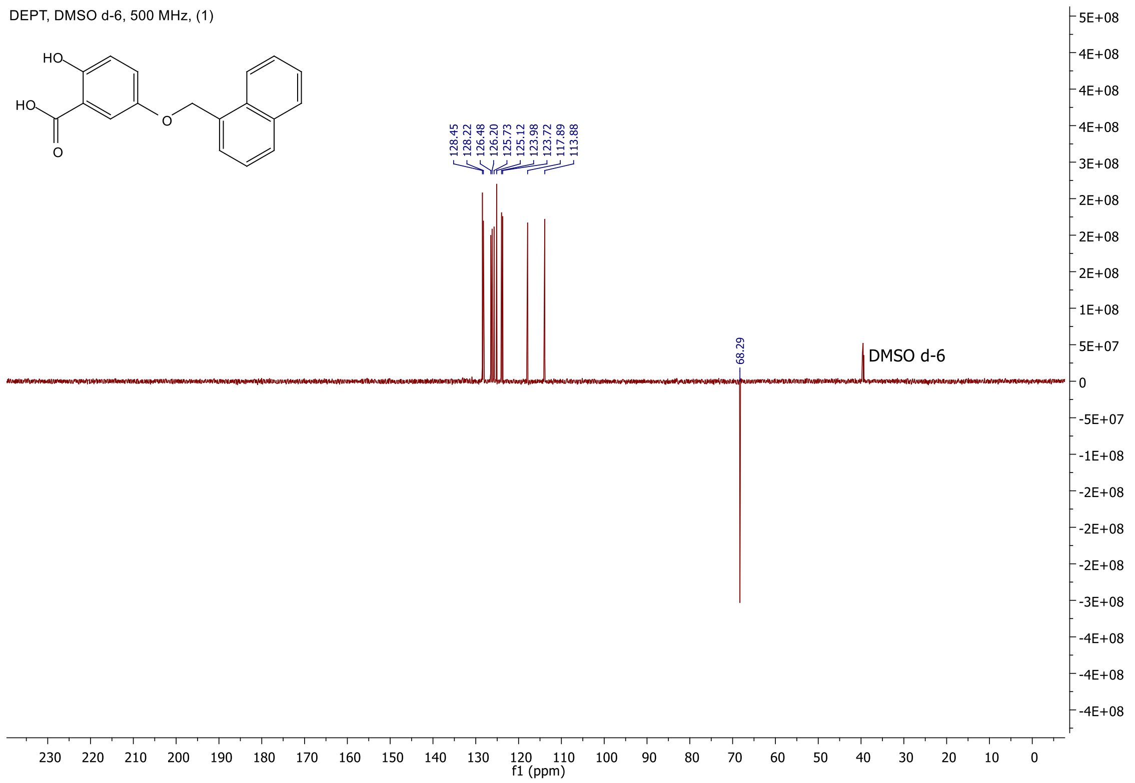
DEPT, DMSO d-6, 500 MHz, (1)
HO
HO
O
O
128.45
128.22
126.48
126.20
125.73
125.12
123.98
123.72
117.89
113.88
68.29
DMSO d-6
f1 (ppm)

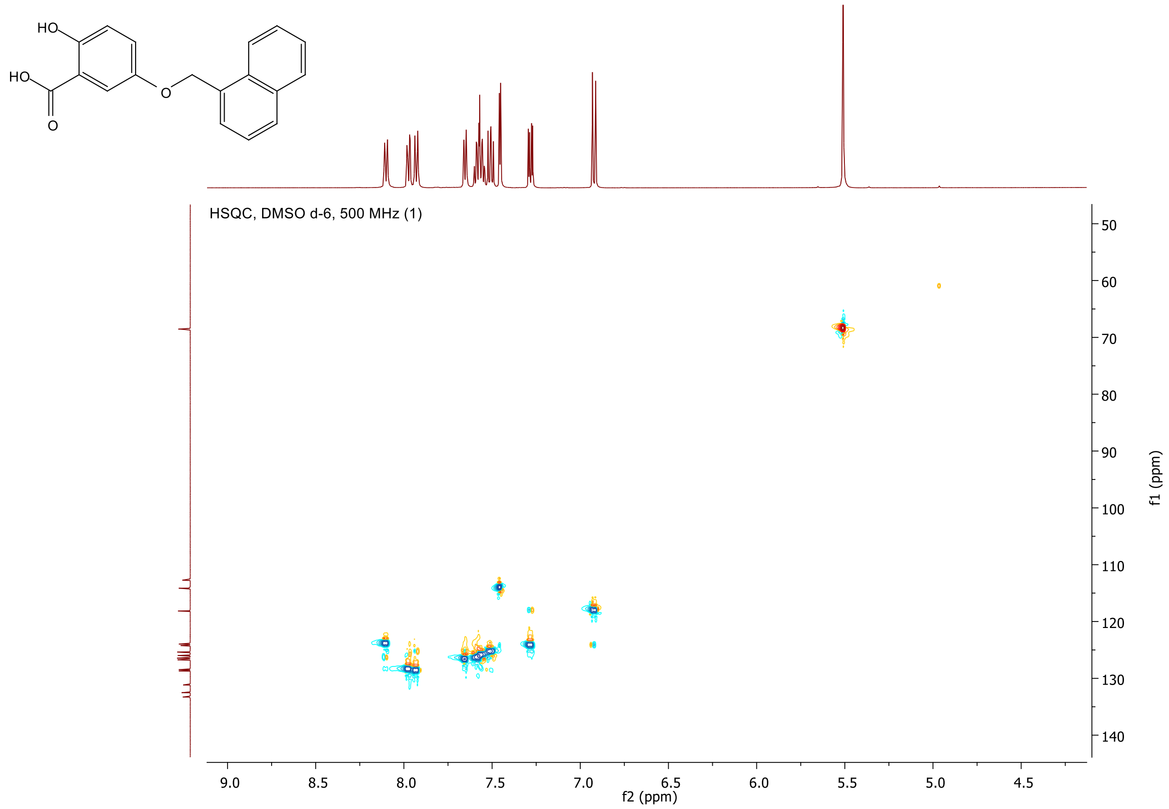
HO
HO
O
O
HSQC, DMSO d-6, 500 MHz (1)
f2 (ppm)
f1 (ppm)

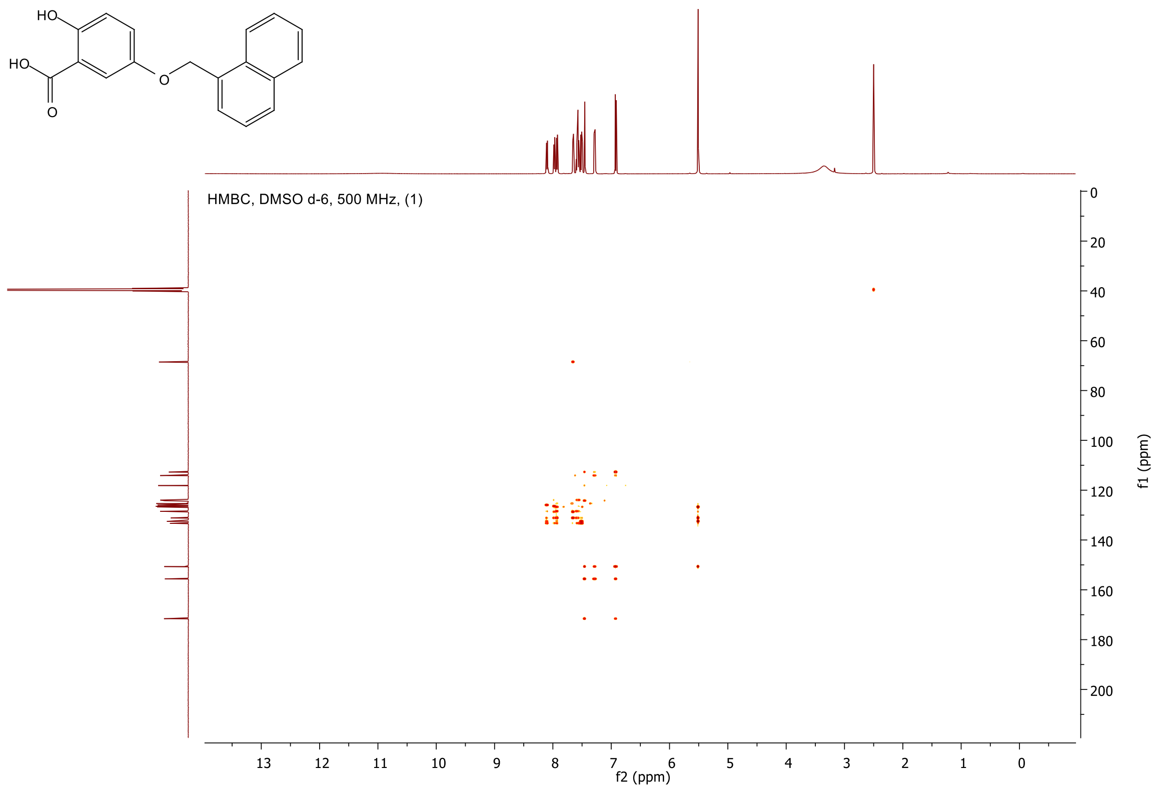

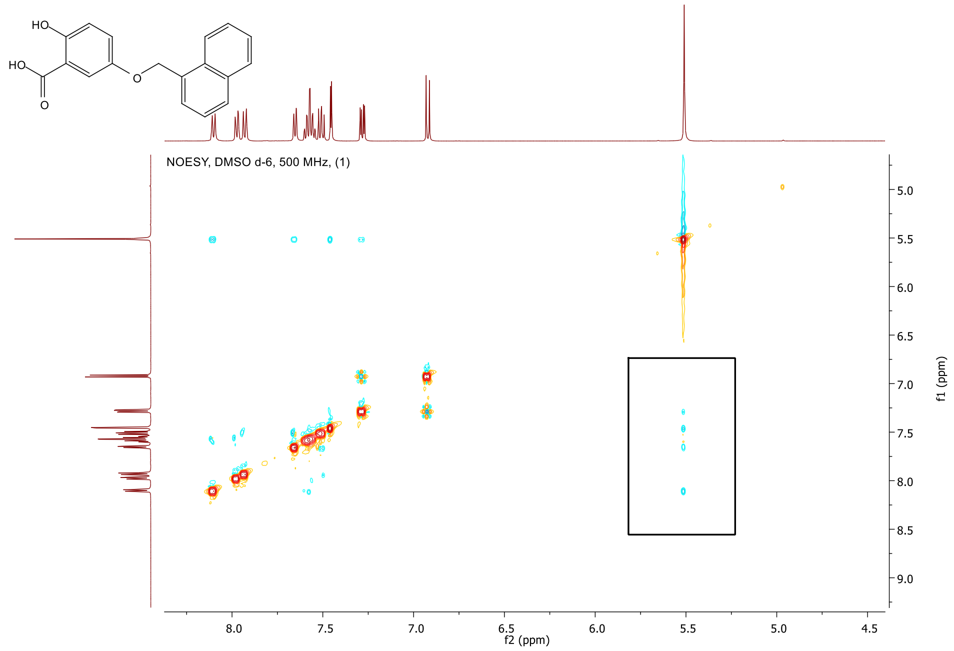

Supplementary Figure 6 ^{1}H-NMR, ^{13}C-NMR, DEPT, HSQC, HMBC, NOESY spectra of compound **1**.

(A)

(B)

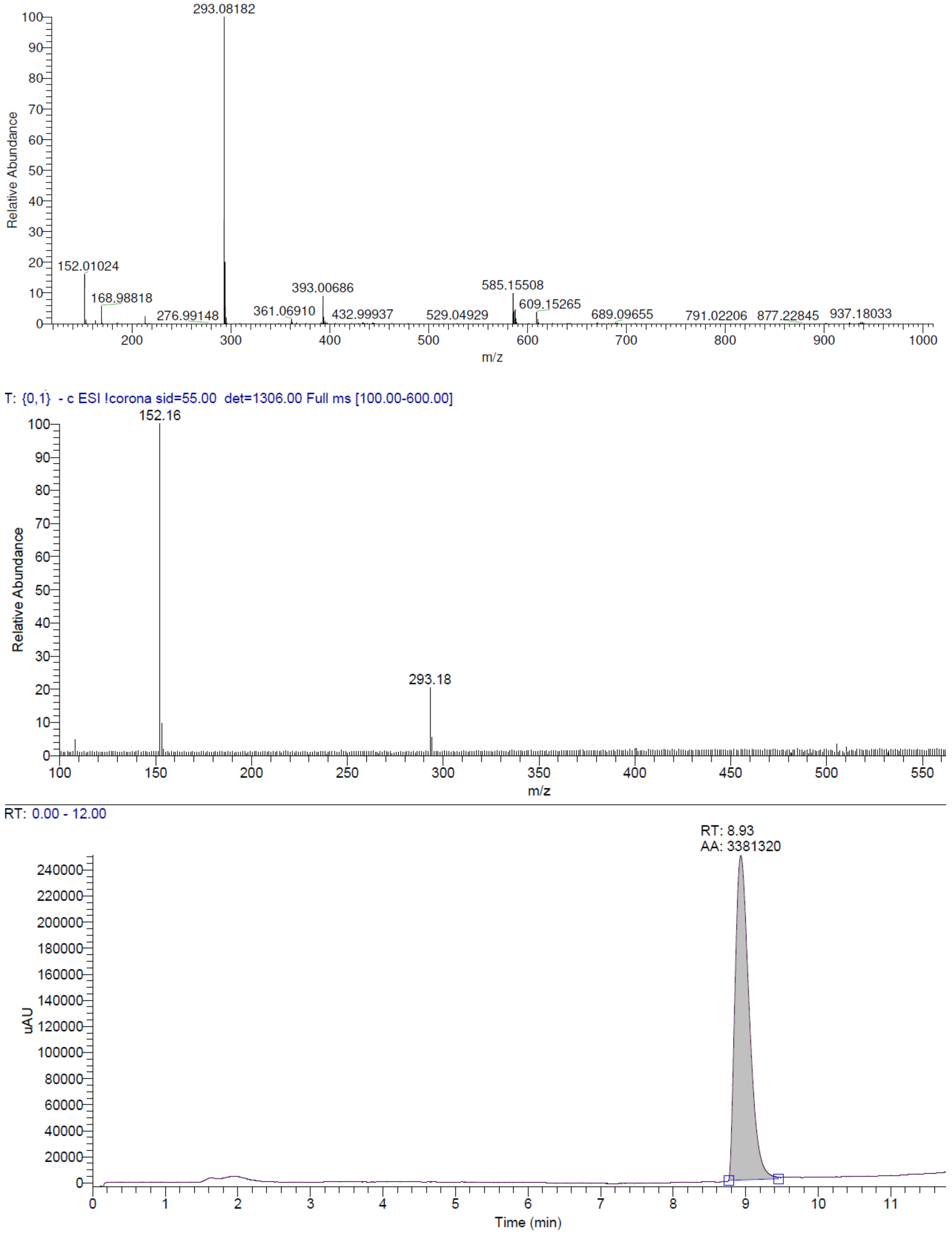

Supplementary Figure 7 (A) Mass spectrum (HR-MS); (B) UV chromatogram (LC-MS) of compound **1**.

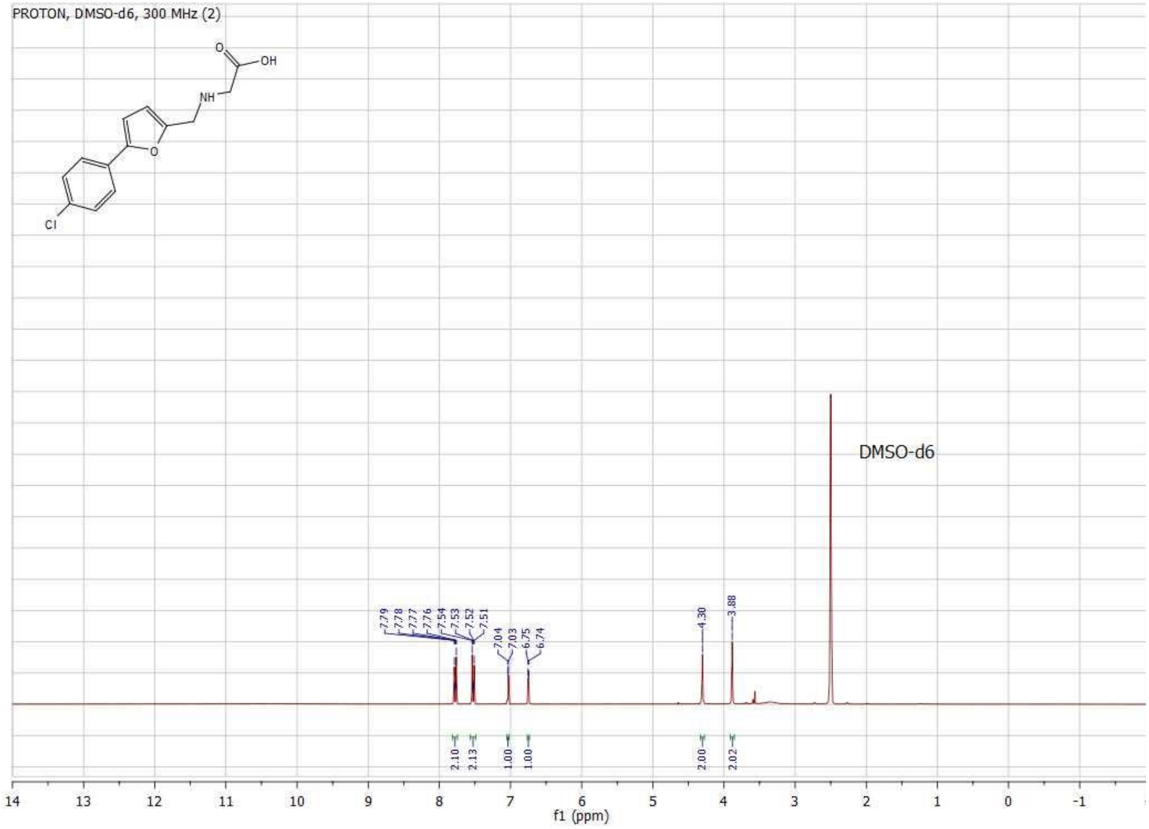

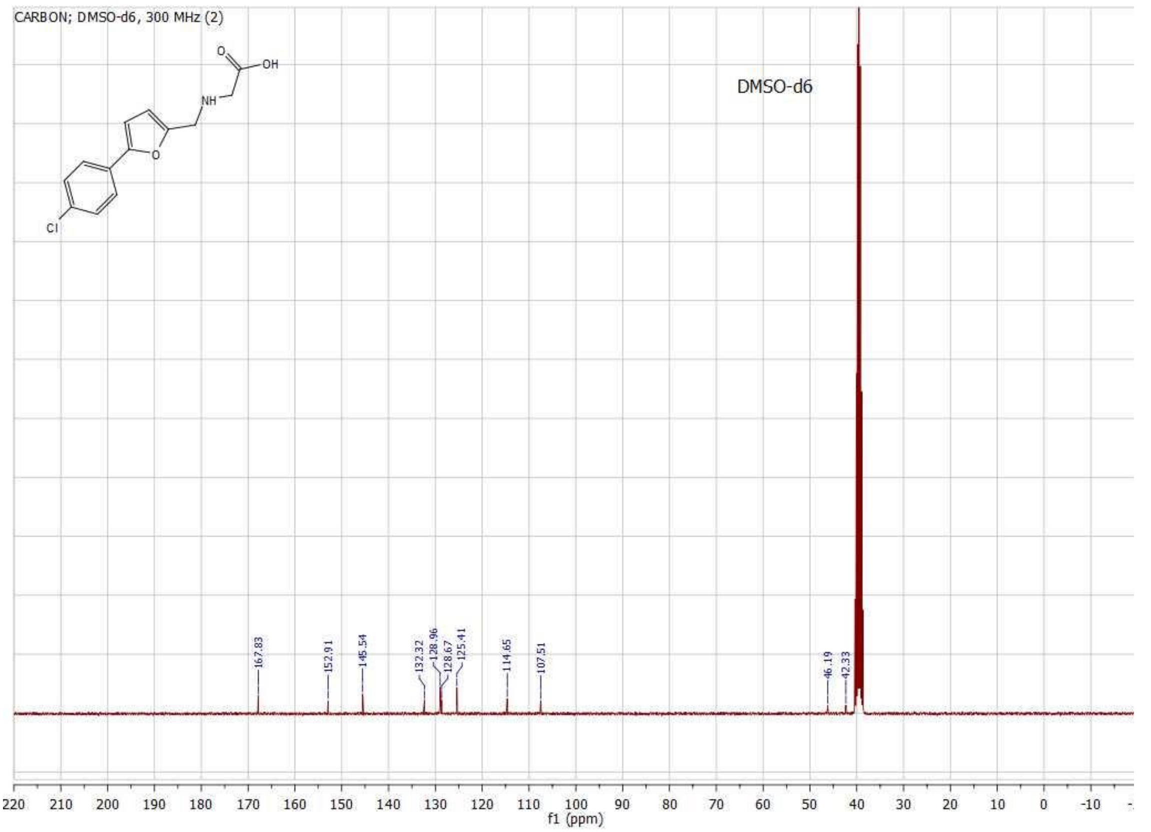

Supplementary Figure 8 ^{1}H-, ^{13}C-NMR spectra of compound **2**.

(A)

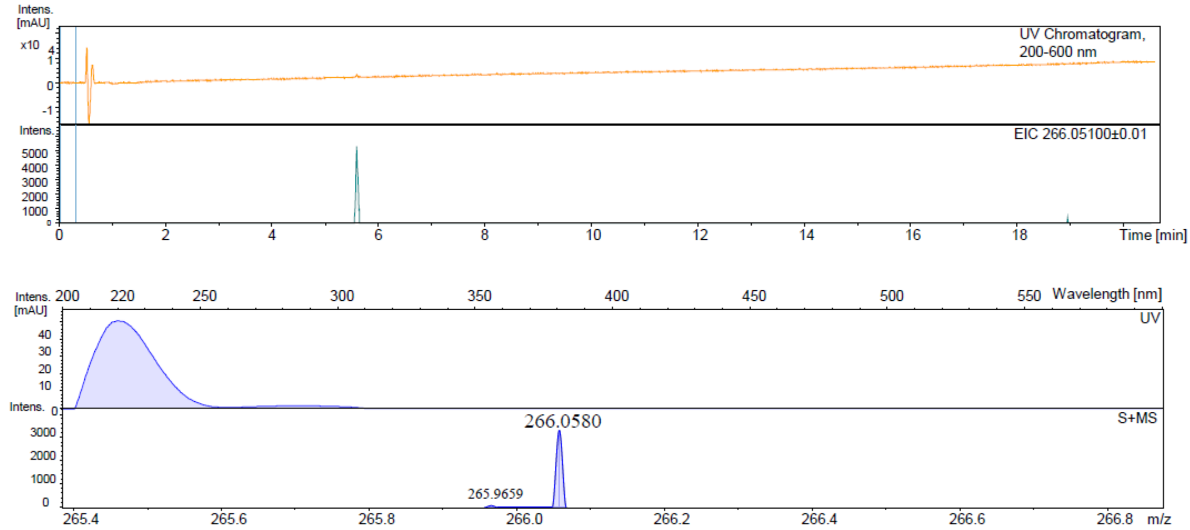

(B)

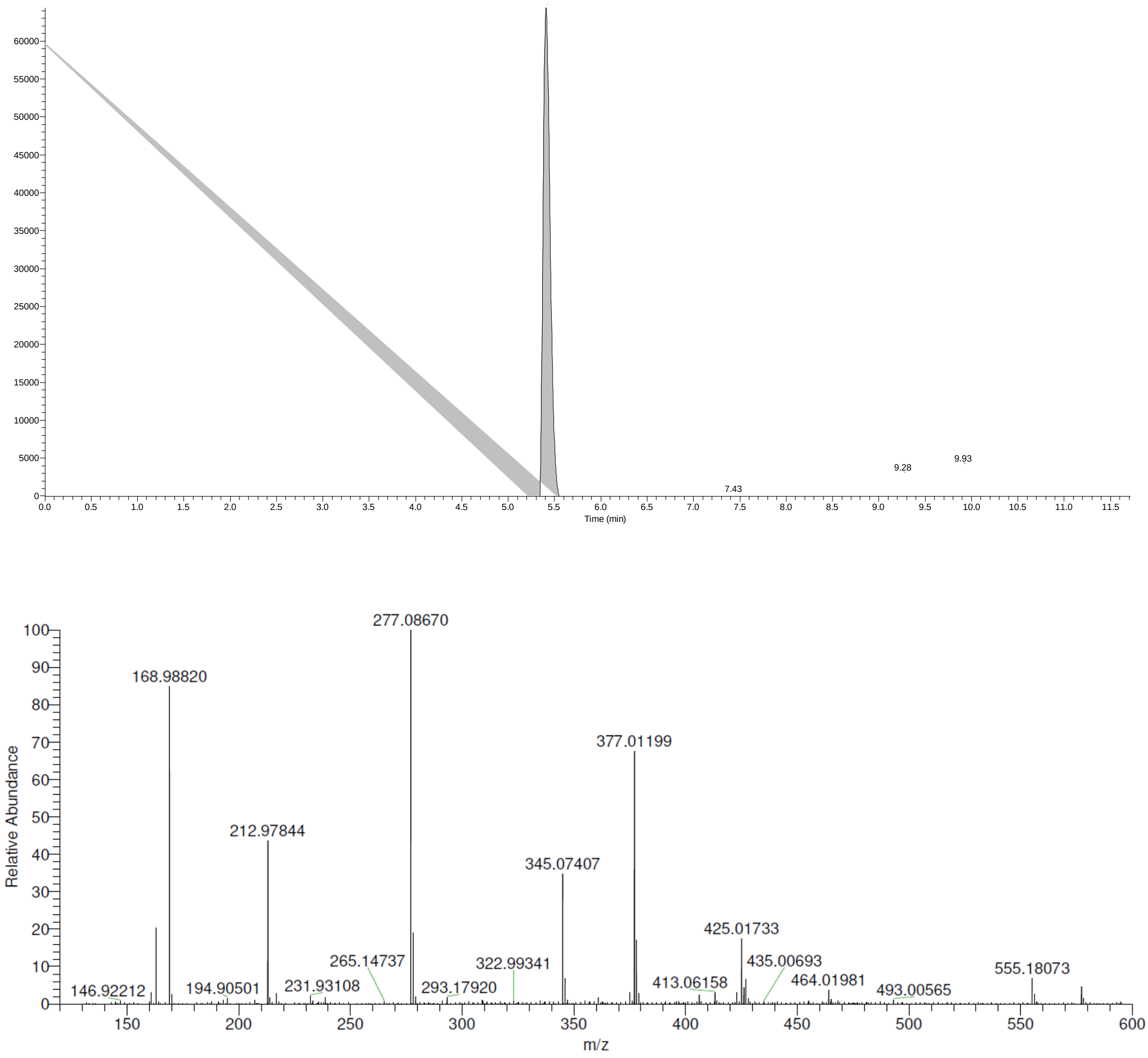

Supplementary Figure 9 (A) Mass spectrum (HR-MS); (B) UV chromatogram (LC-MS) of compound **2**.

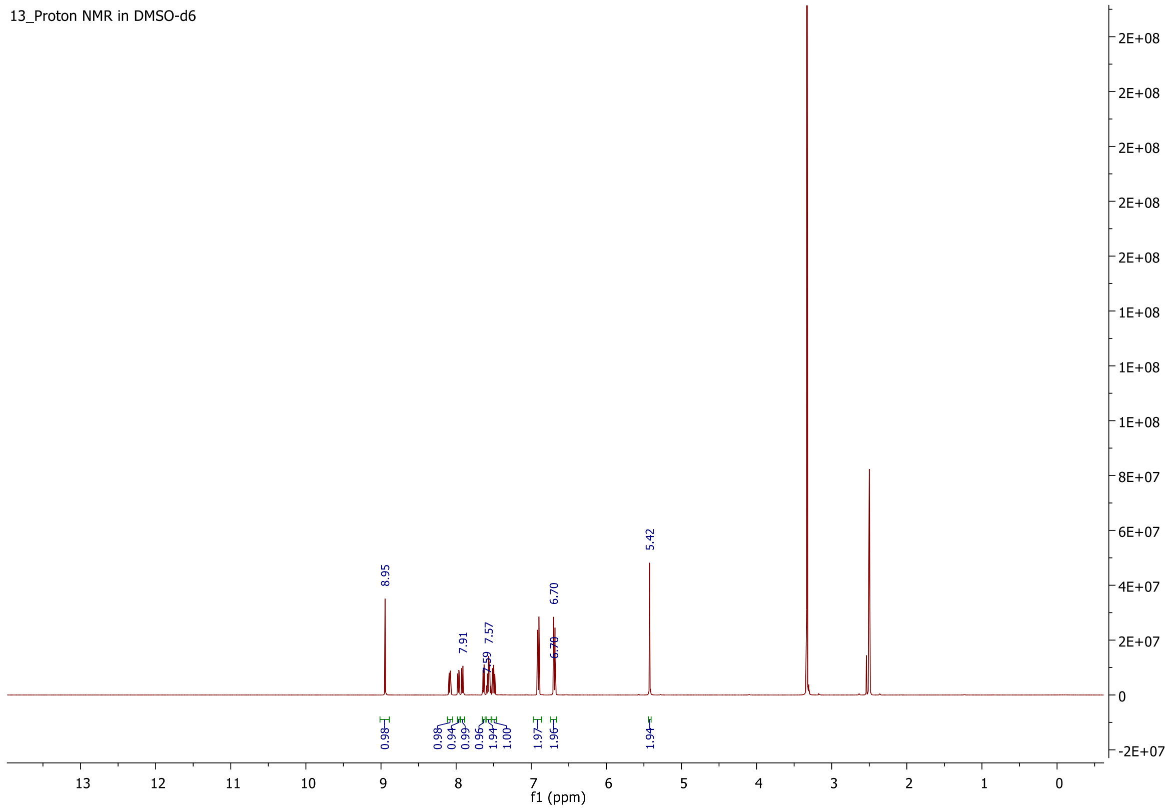

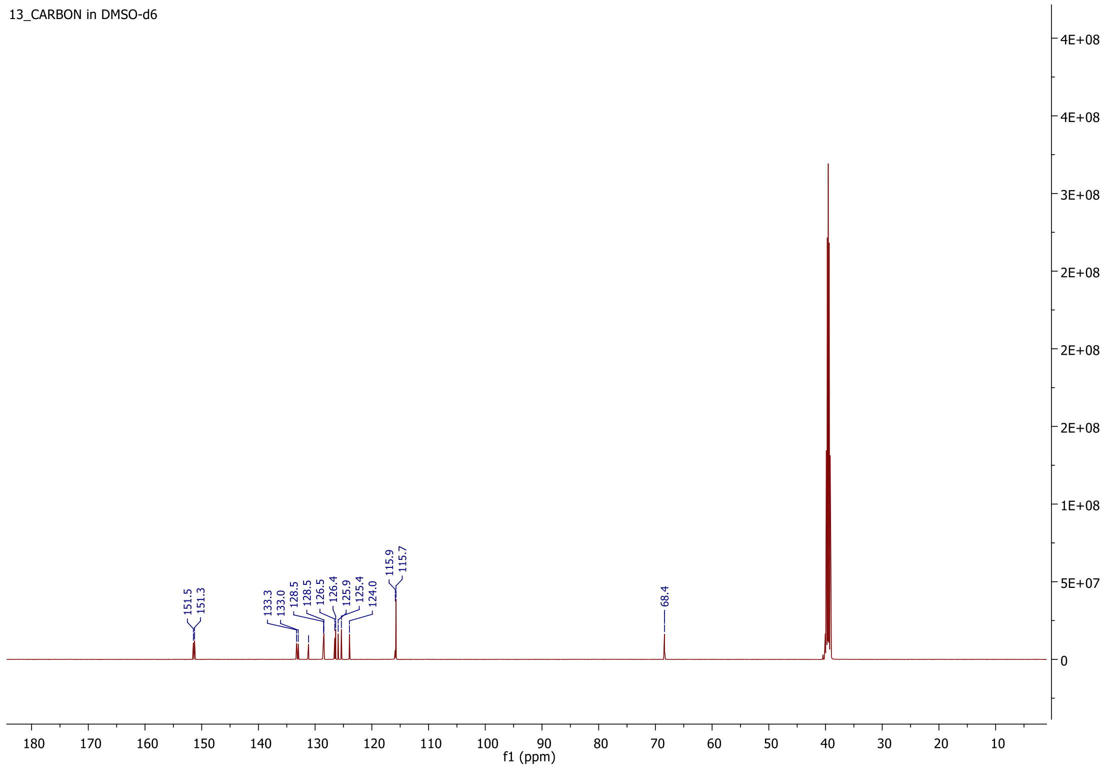

Supplementary Figure 10 ^{1}H-, ^{13}C-NMR spectra of compound **13**.

(A)

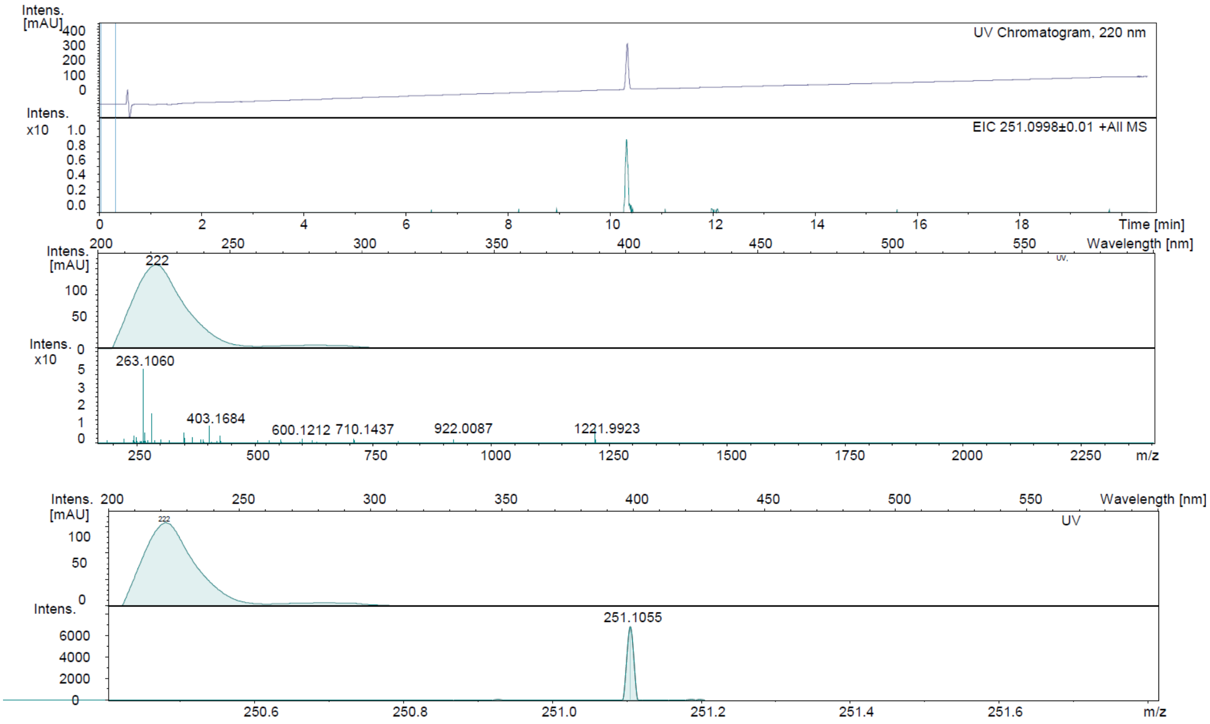

(B)

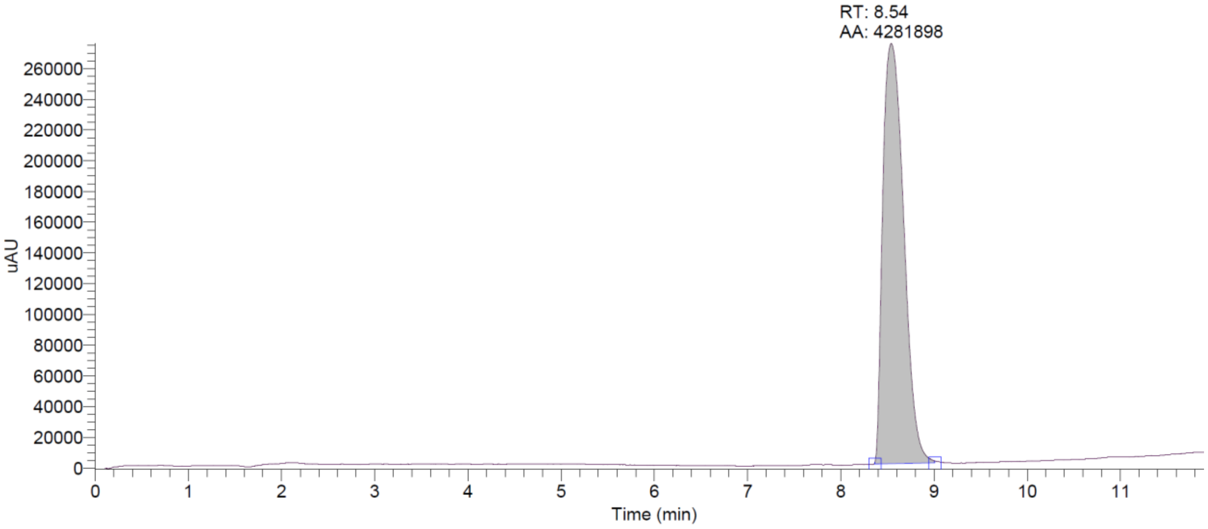

Supplementary Figure 11: (A) Mass spectrum (HR-MS); (B) UV chromatogram (LC-MS) of compound **13**.

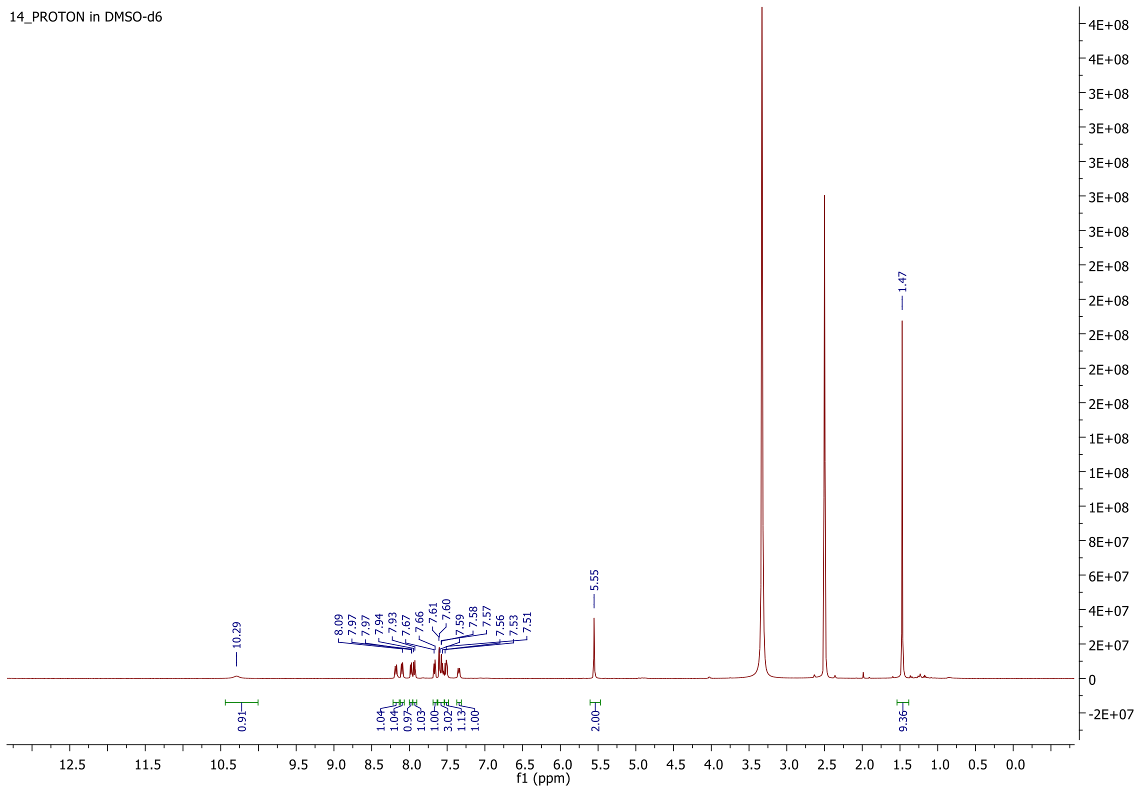

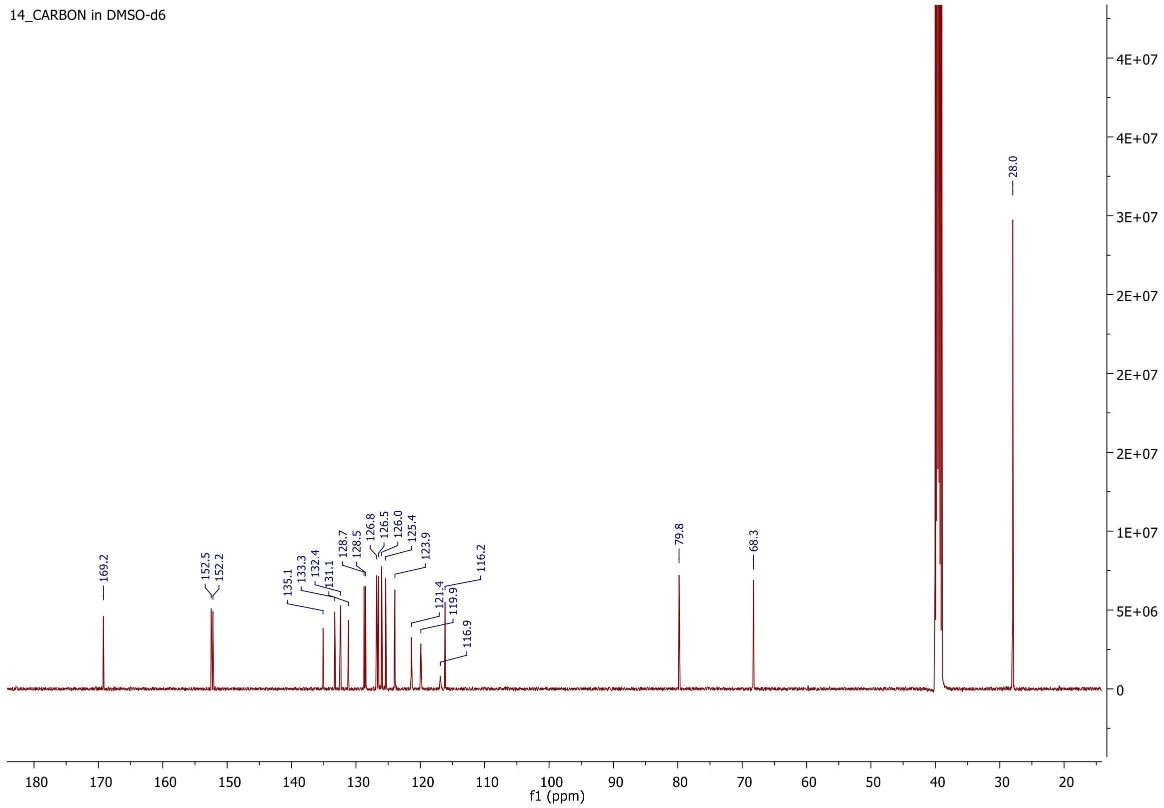

Supplementary Figure 12 ^{1}H-, ^{13}C-NMR spectra of compound **14**.

(A)

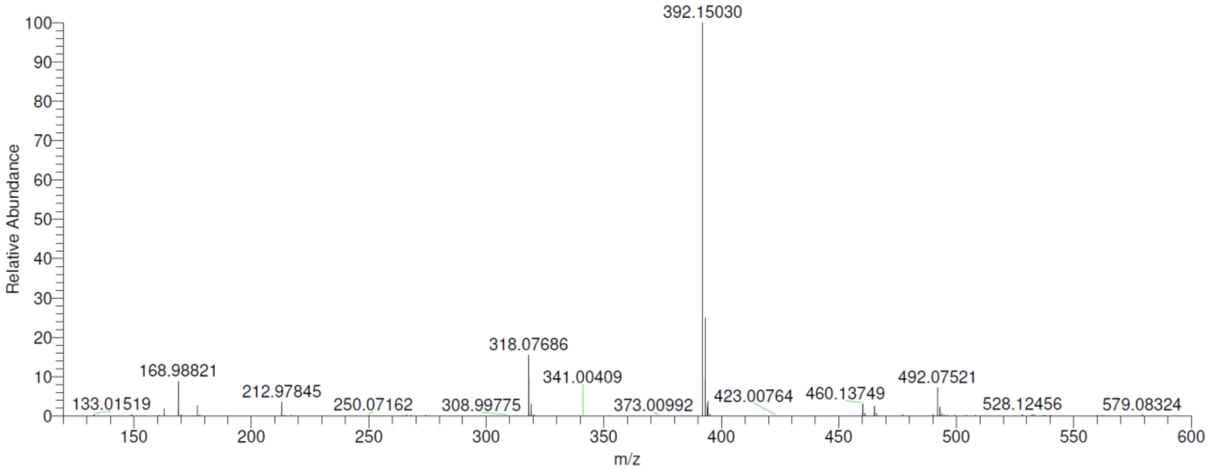

(B)

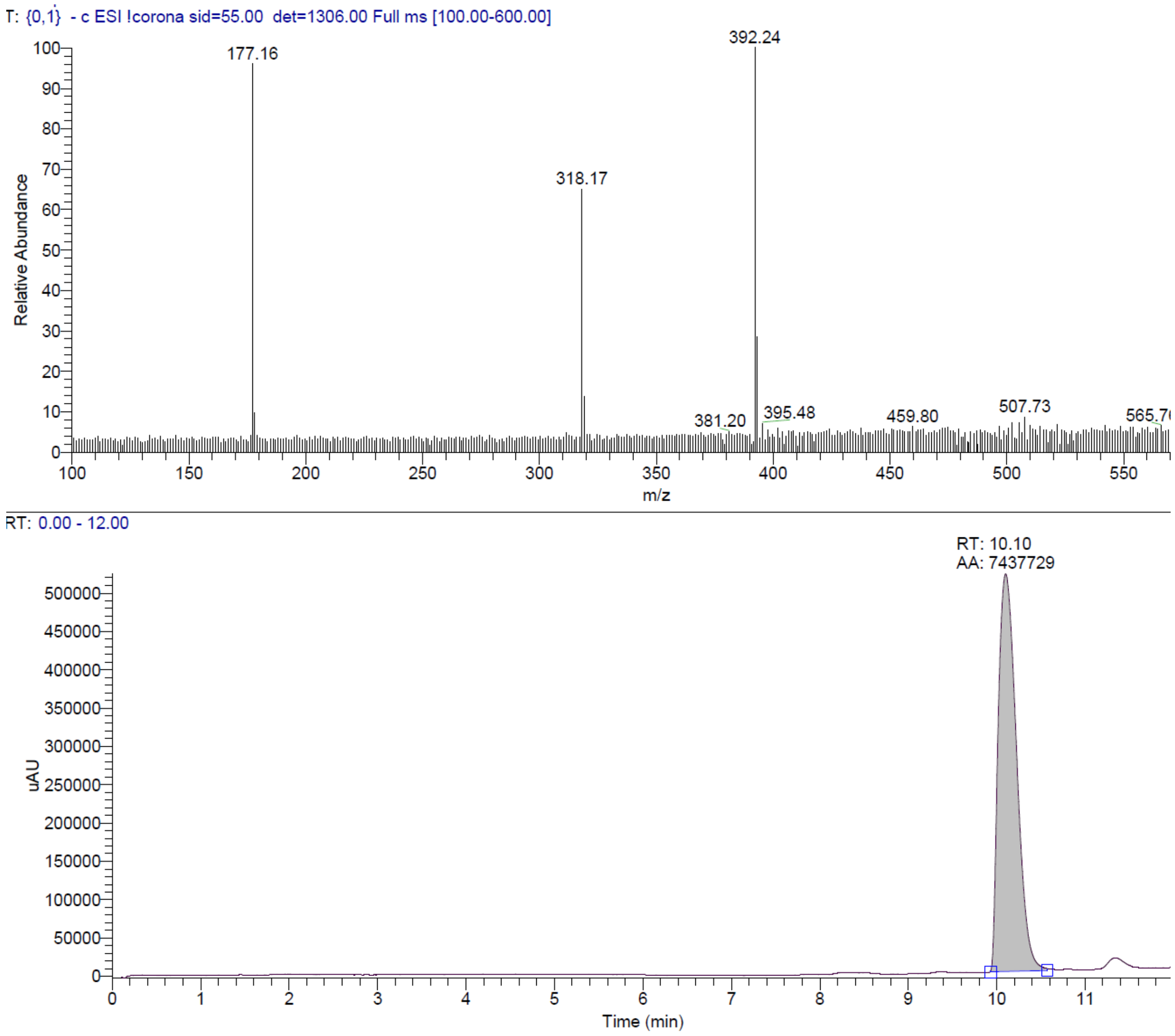

Supplementary Figure 13 (A) Mass spectrum (HR-MS); (B) UV chromatogram (LC-MS) of compound **14**.

Supplementary Notes 5 - Coarse-grained molecular dynamics simulations

Coarse-grained models

All simulations were performed using the new version of Martini 3 Coarse-Grained (CG) force field[17]. The CG protein model was generated using the new version of the program Martinize[18] . The crystallographic structures of the apo form of the ECF FolT2 (PDB code: 5JSZ)[6] and ECF PanT (PDB ID 6ZG3)[19] containing all four subunits were used as references. ATP was not included in the ECF CG models. Elastic networks were applied to each monomer of the ECF complex, with a distance cut-off of 0.85 nm using a force constant of 1300 kJ mol^{-1} nm^{-2}. To further increase stability of the complex, additional harmonic bonds were added between four pair of residues of the ECF complex: Gln96 (chain A)–Leu213 (chain D), Ser172 (chain A)–Gly175 (chain B), Arg257 (chain A)–Asp185 (chain B) and Gly110 (chain B)–Arg179 (chain D). They mimic hydrogen bonds of neighboring monomers. These choices for our ECF CG model are based on our previous work, where we compared the overall flexibility of the ECF complex with atomistic simulations[20]. CG models of compounds **1**, **13** and **14** were obtained according to the parametrization rules of Martini 3, as described [17,21,22]. Bond and angle parameters were parametrized using as reference an atomistic trajectory obtained using the TIP3P [23] water model and OPLS-AA parameters[24,25] generated with LigParGen server[26]. Bond distances were optimized according to the center of geometry mapping (including the hydrogen atoms), which give a good compromise of molecular solvent-accessible surface area and bulk density[17]. The naphthalene ring was built using four TC5 beads connected with constraints, and one central TC5e beads, described as virtual site. The salicylic acid moiety was modeled with two TC5 beads (mimicking 4 aromatic carbon atoms and associated hydrogen atoms), one TP1 (ethanol group) and SQ5n (acetic acid group). The methoxide bridge connecting the rings was represented by a TN2a bead. Improper dihedrals were used to keep the aromatic rings planar. No dihedral was added involving the methoxide bridge, which allows free rotation of one ring in relation to the other one, as expected in an aliphatic ether. Compounds **13** and **14** were built based on the compound **1**, used as template. In compound **13**, the SQ5n bead was removed, with some adaptations on the bond lengths performed to better represent the remaining phenol fragment. On other hand, for compound **14**, the bead TP1 was replaced by three beads: TN6d-SN5a-SC2. These beads better represent the NHBoc group, including the H-donor acceptor character of the –NH (TN6d) and ester group (SN5a), but also the bulky *tert*-butyl hydrophobic group (SC2). All the ligand models are provided in MAD (https://mad.ibcp.fr/) [27] and as part of the supporting material. Lipid models were based on the previous Martini 2 force-field[28,29], but now following the rules for mapping of Martini 3[17] and also with adaptations in the bonded parameters inspired by the "extensible model" of Carpenter et al 2018[30].

System setup and settings of the MD simulations

The ECF transporter was embedded in a bacterial membrane model composed of 1-palmitoyl-2-oleoyl-sn-glycero-3-phosphoethanolamine (POPE), 1-palmitoyl-2-oleoyl-sn-glycero-3-phosphoglycerol (POPG) and cardiolipin (CL) in a ratio of 70:25:5. The simulation box with dimensions of 15 x 15 x 17 nm^3 was built using the INSANE program[29] . The ECF module was positioned in the membrane according to the data derived from the OPM database[31]. The orientation of the principal z-axis of the protein was set to be parallel to the normal of the lipid bilayer. The system was solvated using water solution with 0.15 M concentration of NaCl, mimicking physiological conditions. Ten copies of hit compound were randomly placed in the solvent, which is an equivalent to a water solution of around 8.7 mM. The systems were minimized for a maximum of 10,000 steps with the steepest descent method, followed by a first equilibration of 2 ns, with both steps allowing protein side chains, membrane and the solvent to relax while the backbone was kept fixed using position restraints. The restraints were subsequently removed and the system was equilibrated for another 2 ns. The production simulations were performed for 30 µs. This procedure was repeated 10 times for ECF FolT2 with compounds **1**, **13** and **14**, resulting in a total sampling of 300 µs. The same procedure was considered for ECF PanT with compound **1**. Settings for the CG simulations followed the "new" Martini set of simulation parameters[32]. Temperatures of the system were kept at 310 K with the velocity rescaling thermostat[33,34]. For the pressure, we used the semi-isotropic coupling at 1 bar using the Parrinello-Rahman barostat[35,36] . All simulations were performed with GROMACS (version 2018.8)[37]. All the initial coordinates and backmapped structures are included as part of the Supporting Information.

Analysis of the trajectories

Ligand density was used to determine the main pockets of the compounds observed in the MD simulations. Firstly, the ligands were placed at the minimum distance to the ECF complex in every snapshot with respect to the periodic boundary conditions. Then the ECF structure was positioned in the box center and its backbone was aligned with the crystal structure. The ligand density was obtained by computing the occupancy of the ligand in the three-dimensional space using the Volmap plugin of VMD[38]. The grid points had a distance of 0.2 nm. All ligand bead sizes were taken into account. Binding free energies (ΔG_{bind}) were estimated based on the populations of the ligand in the pockets (p_{pocket}, considering to P2, P9 or P11 of ECF FolT2 and the single pocket of ECF PanT) and environments ($p_{environment}$, considering water or membrane), according to equation:

$$\Delta G_{bind} = -RTln\left(\frac{10 \times p_{pocket}}{p_{environment}}\right)$$

The ratio p_{pocket} / $p_{environment}$ was multiplied by a factor of 10, considering that the simulations were performed with 10 molecules of compound **1** in the simulation box. In such condition, the $p_{environment}$ would be higher as no more than one ligand can occupy the pockets while the remaining molecules can stay in the environments. In addition, a correction that accounts

for the change in the ligand's translational volumes (for pocket-water) or area (for pocket-membrane) was also included in the estimate of ΔG_{bind} [39]. The states were defined based on the normalized number of contacts of the ligand with the pockets, water and membrane (plots of Supplementary Figures 14 and 15), using a cutoff of 0.55 nm between the beads of the ligand and the residues of the ECT complex. The number of contacts was computed using GROMACs tool mindist. Errors were estimated with block average approach. The representative poses of compounds **1** and **14** in P9 were backmapped from the CG to the atomistic resolution using the backward program[40]. Binding modes that showed high frequency and also were on the center of ligand density, were chosen as representative poses for this work. To quantify the importance of hydrogen bonds, the total time which a ligand stays in contact with a certain pocket was compared with the time the ligand stayed in contact with residues Ser173 and Lys102 in the S-component of ECF-FolT and Arg76 of the S-component of ECF-PanT.

A–B	Compound 13		Compound 1		Compound 14	
	$\Delta G_{A\text{-}B}$ (kJ/mol)	Error (kJ/mol)	$\Delta G_{A\text{-}B}$ (kJ/mol)	Error (kJ/mol)	$\Delta G_{A\text{-}B}$ (kJ/mol)	Error (kJ/mol)
P9 – water	-16.09	±1.24	-18.80	±0.81	-20.37	±0.36
P9 – lipids	8.21	±1.41	-1.48	±0.79	0.17	±0.13
P11 – water	-18.32	±0.51	-13.12	±0.44	-16.42	±0.58
P11 – lipids	5.99	±0.59	4.21	±0.42	4.12	±0.35
P2 – water	≥ 0.72	-	-11.60	±1.11	-8.95	±6.25
P2 – lipids	≥ 25.02	-	5.73	±1.13	11.58	±6.02
lipids – water	-18.97	±0.53	-12.00	±0.10	-15.20	±0.23

Supplementary Table 1. Binding free energies and lipids/water partitioning free energies estimated of compounds **1**, **13**and **14** in relation to the pockets (P2, P9 or P11) and environments (water or lipids). Binding of compound **13** to P2 was not observed, with the upper limited estimated by total sampling of the simulations. Number of binding events of compound **14** to P2 was reduced, which result in the large error in this particular case.

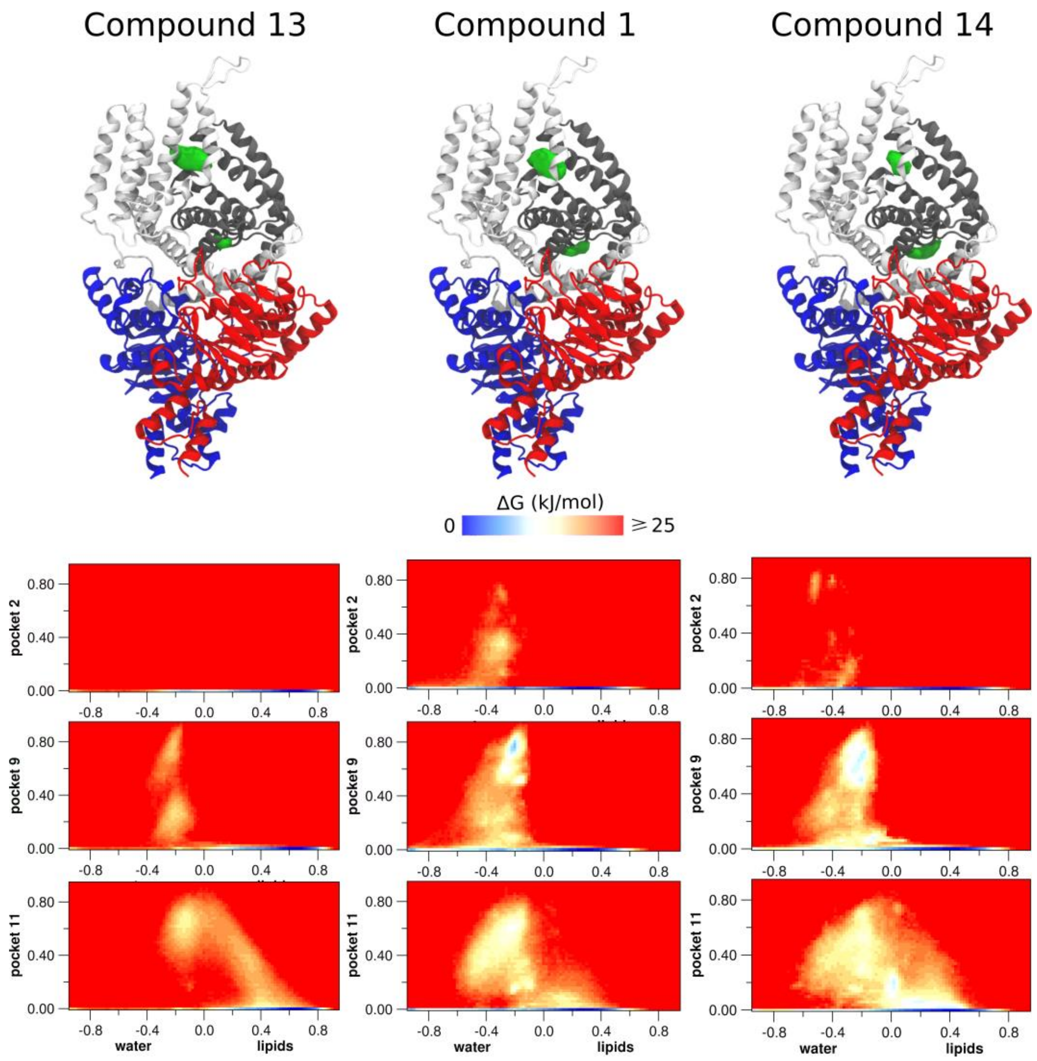

Supplementary Figure 14. On top of the figure, the ligand density of compounds **13** (left), **1** (center) and **14** (right) obtained from the simulations are shown in green, together with crystal structure of ECF FolT2 (PDB ID 5JSZ), in complex with EcfA and EcfA' shown in blue and red, EcfT in white and S-component in gray. On the bottom of the figure, plots of free energy landscapes spanned by the normalized contacts of the compounds with the pockets P2, P9 and P11 and by the normalized contacts with the lipids subtracted by the normalized contact with the water. The colour scale indicates the free energies values, from blue (0 kJ/mol), passing by white (12.5 kJ/mol) until red (≥ 25 kJ/mol). The free energies in the plots were not normalized by the ligand concentration or volume/area terms.

A–B	Compound 1	
	$\Delta G_{A\text{-}B}$ (kJ/mol)	Error (kJ/mol)
pocket– water	−12-6	±1.4
pocket– lipids	4.6	±1.3
lipids – water	−11.9	±0.1

Supplementary Table 2. Binding free energies and lipids/water partitioning free energies estimated of compounds **1** in relation to the pocket of ECF-PanT and environments (water or lipids).

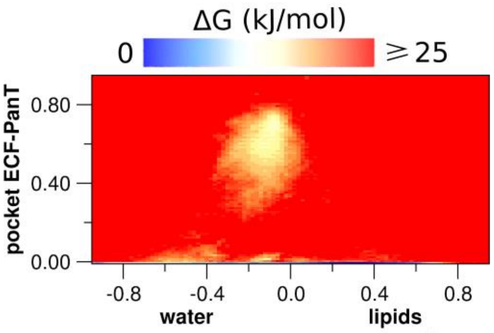

Supplementary Figure 15. Free energy landscape plot spanned by the normalized contacts of the compound 1 with the pocket observed in ECF-PanT and by the normalized contacts with the lipids subtracted by the normalized contact with the water. The colour scale indicates the free energies values, from blue (0 kJ/mol), passing by white (12.5 kJ/mol) until red (≥ 25 kJ/mol). The free energies in the plots were not normalized by the ligand concentration or volume/area terms.

Supplementary Notes 6 - Docking Studies in SeeSAR

In order to understand the difference between the dockings into a rigid conformation of the protein and the flexible site using the MD simulation, we redocked the compounds **1**, **13** and **14** into the P2, P9 and P11 pockets. We used this time the relaxed protein conformation that we generated thought the MD simulations. The compounds were docked using the version of SeeSAR 10.3.1. To compare the results directly with the MD simulation, we focused our efforts towards the same compounds used in the studies above including **1**, **13** and **14** (Supplementary Table 3). Following the *in vitro* data, we would expect **14** showing the best estimated affinity close to low µM range followed by the initial hit compound **1**. Lastly compound **13**, as negative control should exhibit the worst estimated affinity, potentially in mM range or even higher. At all cases, we observed that the docking results do not correlate with the *in vitro* data or the proposed MD simulations. Specifically, in P2 pocket, we observed that compounds **13** has the best estimated affinity at low µM range whereas compound **1** is estimated to be in low mM range and the most promising compound **14** according to biochemical data, does not seem to fit in the pocket at all. Continuing with the pockets P9 and P11, we observed a similar pattern with both compounds **1** and **13** exhibiting similar ranges of estimated affinities to low µM levels. However, compound **14** seems to fit on both these pockets but with a lower affinity close to mid µM range.

A–B	Estimated affinity (µM)		
	Compound 13	Compound 1	Compound 14
P9	13,6	9	99,7
P11	2,8	8,4	9,3
P2	56	36000	100000>

Supplementary Table 3. Binding free energies and estimated affinities of compounds **1**, **13** and **14** in relation to the pockets (P2, P9 or P11). Binding of compound **14** to P2 pocket was not observed. In all the pockets, negative control compound **13** exhibits estimated affinities at similar range with the initial hit compound **1**.

To justify the difference between the MD simulations and the dockings, we examined carefully both pdb structures used. One was generated with the MD simulations having a relaxed conformation with minimized energy while the second was the original structure of ECF-FolT2 (PDB code 5JSZ). We aligned and inspected both structures only to find out that there is a significant movement of the relaxed structure compare with the original one. This movement differentiates between the helices over the whole protein (Supplementary Figure 16A). Specifically, upon inspection of the P2 pocket, we observed that the movement of some helices of the original structure highlighted with dark red color are moved into a position at the relaxed structure that hinder the entrance of the compounds into the pocket from the front side. Although the pocket is still accessible from another side, it changes its conformation. This change to the 3D structure of the P2 pocket, explains the results from the MD simulations that indicate the compounds having a better and a more accessible pocket to bind to than the P2 pocket (Supplementary Figure 16B).

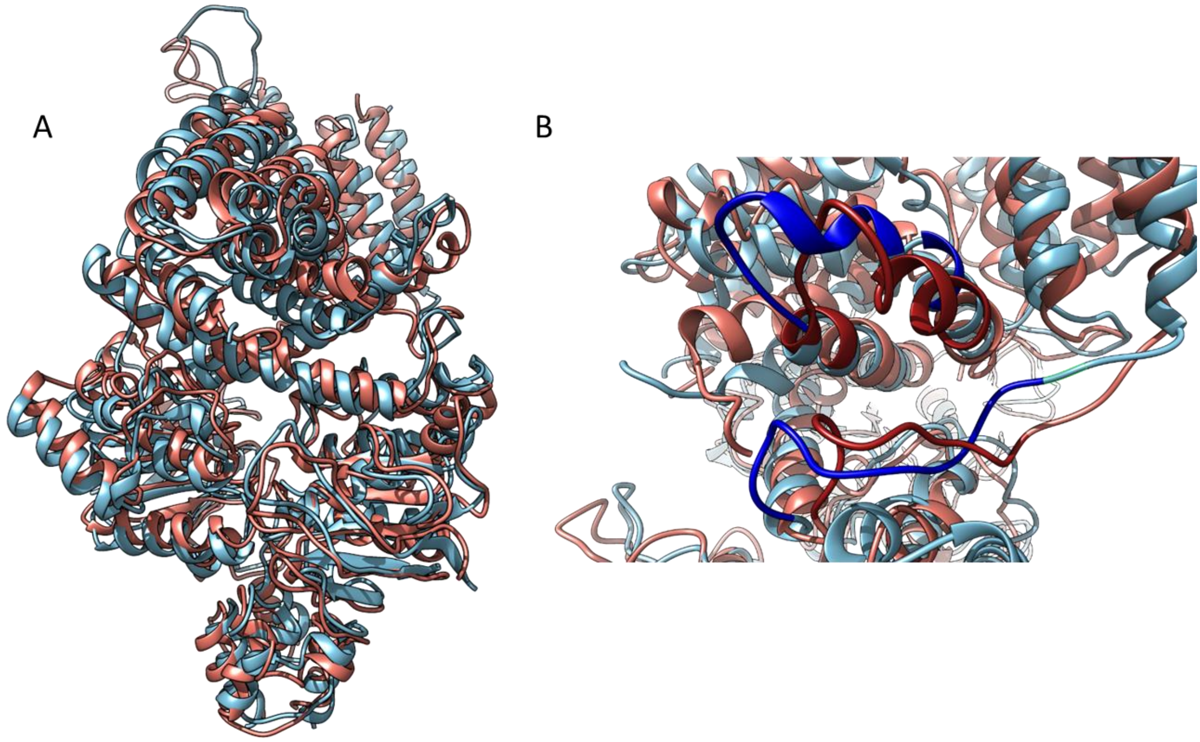

Supplementary Figure 16. Aligned structures of ECF FolT2 transporter depicted in ribbons. The original structure (PDB code 5JSZ) is colored in light blue whereas the relaxed structured with minimized energy obtained from the MD simulation is colored in red. (A) Whole complex of the ECF FolT2 protein. (B) P2 pocket of the ECF FolT2 protein. The helixes with the most substantial movement from both structures are highlighted with dark blue and with dark red for the original and the relaxed structure, respectively.

Supplementary Note 7 - Site-directed mutagenesis

Mutagenesis

Lysin 102 in FolT2 was replaced by a negatively charged residues Aspartic acid (Asp) or Glutamic acid (Glu), which may impact the interactions with the carboxylic group we have in many of the compounds. For this purpose, we cloned the gene encoding FolT2 in the plasmid paCYCara, which is a derivative of pLEMO where the rhamnose promoter was replaced by an arabinose promoter[41].

We designed primers with the desired mutation in the gene encoding FolT2 specified below.

Primer name (mutation)	Primer sequence (5'→3')
Forward ECF-FolT2 K102 to D<br>Reverse ECF-FolT2 K102 to D	gtacGaCcagcccatccagatttg<br>gctgGtCgtacaagaagaagc
Forward ECF-FolT2 K102 to E<br>Reverse ECF-FolT2 K102 to E	gtacGagcagcccatccagatttg<br>gctgctCgtacaagaagaagc

For mutagenesis, we performed a PCR reaction, employing the FolT2 DNA template within the paCYCara[19]. Subsequently, the PCR product underwent digestion with Dpnl enzyme. Following this, the treated product was introduced into *E.coli* MC1061 competent cells using established transformation protocols. Following successful sequencing confirmation by the Genomic Services of Eurofins Genomics, we proceeded with the subcloning of the mutated paCYCara FolT2 fragment into the P2bad vector. This task was accomplished through the utilization of XbaI and XhoI restriction enzymes. Agarose gel electrophoresis was carried out and the fragments of interest were extracted utilizing a DNA extraction kit provided by QIAGEN. Subsequently, we performed ligation using the DNA extract and P2bad plasmid[6] and finally, we transformed them into *E. coli* competent cells.

Expression, purification and reconstitution of ECF-FolT2 mutants in proteoliposomes and radiolabeled uptake assay.

The mutants of ECF-FolT2 from *Lactobacillus delbrueckii* subsp. *bulgaricus* were expressed, purified and reconstituted in liposomes using same procedures, described in Supplementary Note 3. The mutants were purified successfully (Supplementary Figure 17) and all four subunits were present in the SDS-PAGE gel (Supplementary Figure 18).

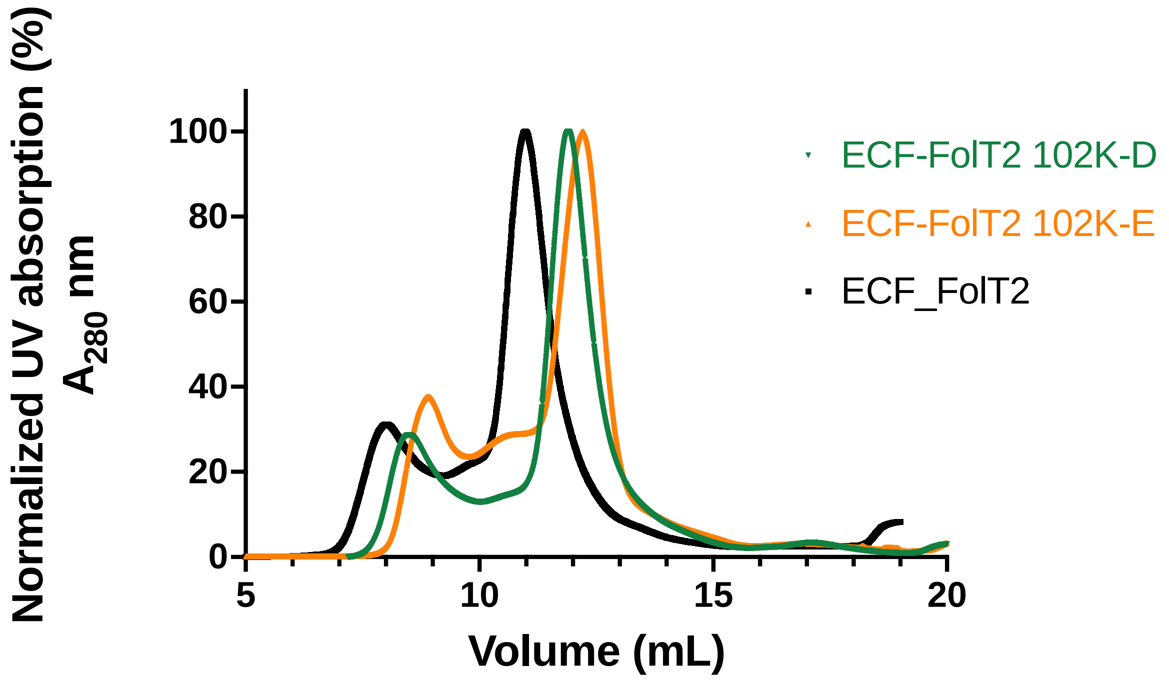

Supplementary Figure 17 Size Exclusion Chromatogram of purifications of ECF-FolT2 (black), and the mutants ECF-FolT2 K102D (green) and ECF-FolT2 K102E (orange).

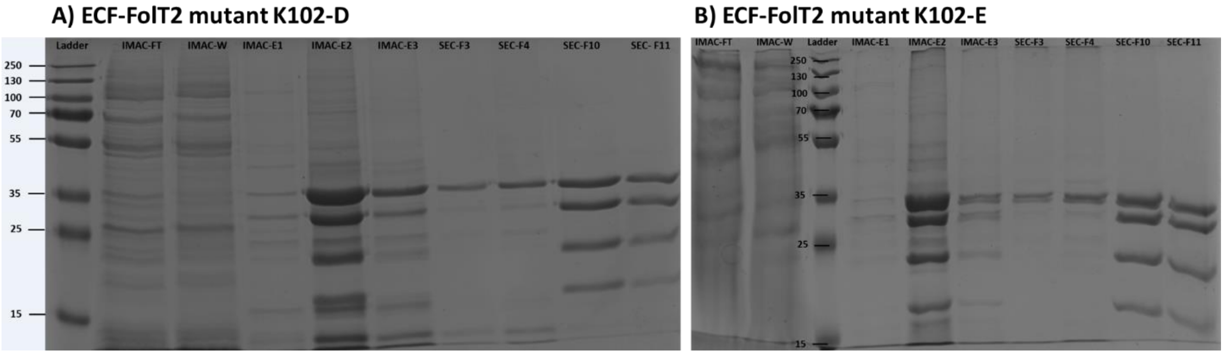

Supplementary Figure 18: Purification of ECF-FolT2 mutants (A) K102D and (B) K102E. Abbreviations in the figures are L: ladder, FT: flow through IMAC columns, W: wash and E1-3 Elution 1, 2, and 3 from IMAC purification. SEC- F3, F4, F10 and F11, Fractions 3, 4, 10, and 11 of the size exclusion chromatography step.

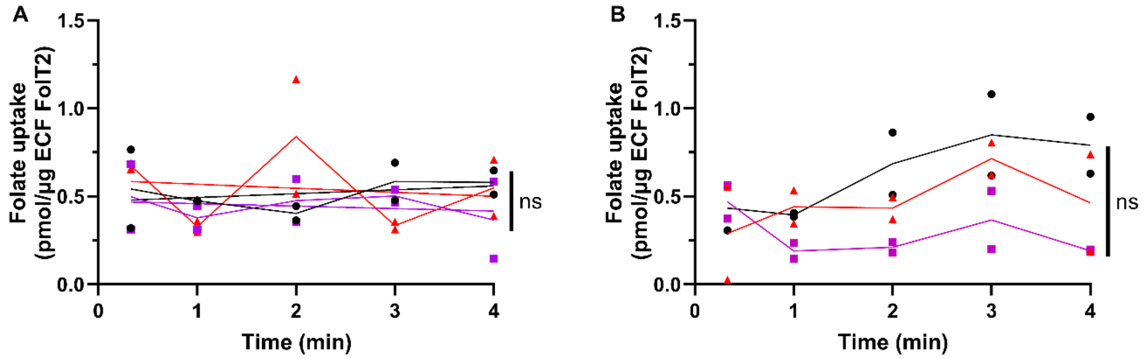

Supplementary Figure 19. Folate uptake by ECF FolT2 mutants and effect of compound 1. (A) K102D mutant reconstituted in liposomes filled with 10 mM of Mg-ATP (black), 10 mM of Mg-ATP and 250 µM of compound **1** (red) and 10 mM Mg-ADP (purple). (B) K102E mutant reconstituted in liposomes filled with 10 mM of Mg-ATP (black), 10 mM of Mg-ATP and 250 µM

of compound **1** (red) and 10 mM Mg-ADP (purple). All samples contained final DMSO concentration of 5% (v/v) in lumen and outside of proteoliposomes and compounds not only in the lumen but also outside of proteoliposomes. Data points on graphs represents individually for two independent measurements. ns is for not significant.

The Figure 20 is the reference for the mutant experiment.

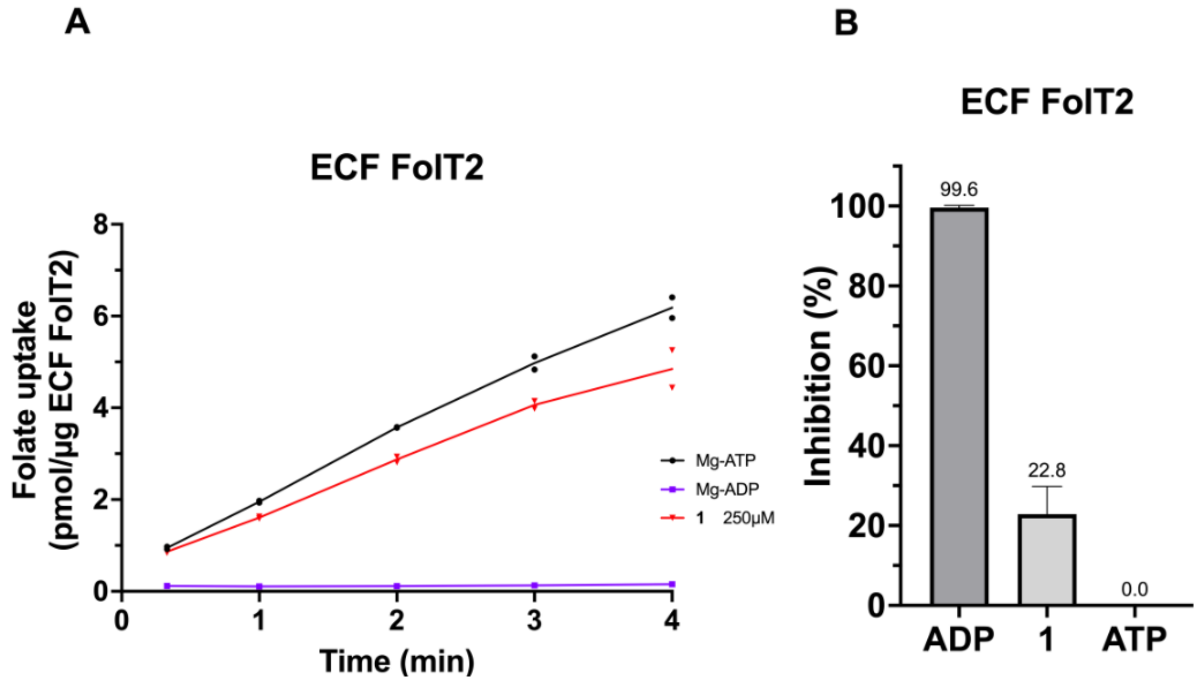

Supplementary Figure 20. Effect of compound 1 on activity of WT ECF FolT2. (A) Folate uptake of proteoliposomes filled with 10 mM of Mg-ATP (red), 10 mM of Mg-ATP and 250 µM of compound **1** (black) and 10 mM Mg-ADP (blue). (B) Relative inhibition rate of WT ECF FolT2 proteoliposomes filled with 10 mM of Mg-ATP, 10 mM of Mg-ATP and 250 µM of compound 1 and 10 mM Mg-ADP. All samples contain final DMSO concentration of 5% (v/v) in lumen and outside of proteoliposomes and compound outside of proteoliposomes. ECF-FolT2 was purified and reconstituted in liposomes at a protein to lipid ratio of 1:250 (w/w). Data points on graphs represents individually for two independent measurements.

The results of the uptake assays suggest that Lysine 102 is an important residue for the activity of the ECF transporter. The mutation of Lysine 102 to aspartic acid or glutamic acid abolished the activity of the transporter, as the uptake traces have not significant difference with the negative control (proteoliposomes filled with Mg-ADP).

Supplementary References

1 Volkamer, A., Griewel, A., Grombacher, T. & Rarey, M. Analyzing the topology of active sites: on the prediction of pockets and subpockets. *J Chem Inf Model* **50**, 2041-2052, doi:10.1021/ci100241y (2010).

2 Volkamer, A., Kuhn, D., Rippmann, F. & Rarey, M. DoGSiteScorer: a web server for automatic binding site prediction, analysis and druggability assessment. *Bioinformatics* **28**, 2074-2075, doi:10.1093/bioinformatics/bts310 (2012).

3 Bousis, S., Setyawati, I., Diamanti, E., Slotboom, D. J. & Hirsch, A. K. H. Energy-Coupling Factor Transporters as Novel Antimicrobial Targets. *Advanced Therapeutics* **2**, doi:10.1002/adtp.201800066 (2019).

4 Hilbig, M. & Rarey, M. MONA 2: A Light Cheminformatics Platform for Interactive Compound Library Processing. *J Chem Inf Model* **55**, 2071-2078, doi:10.1021/acs.jcim.5b00292 (2015).

5 Hilbig, M., Urbaczek, S., Groth, I., Heuser, S. & Rarey, M. MONA - Interactive manipulation of molecule collections. *J Cheminform* **5**, 38, doi:10.1186/1758-2946-5-38 (2013).

6 Swier, L. J., Guskov, A. & Slotboom, D. J. Structural insight in the toppling mechanism of an energy-coupling factor transporter. *Nat Commun* **7**, 11072, doi:10.1038/ncomms11072 (2016).

7 Berthold, M. R. *et al. Stud. Classif. Data Anal. Knowl. Organ.*, (Springer, 2007).
8 GmbH, B. *KNIME Interfaces*, https://www.biosolveit.de/KNIME/).
9 LeadIT version 2.1.8 (2014).
10 Baell, J. B. & Holloway, G. A. New substructure filters for removal of pan assay interference compounds (PAINS) from screening libraries and for their exclusion in bioassays. *J Med Chem* **53**, 2719-2740, doi:10.1021/jm901137j (2010).
11 Bruns, R. F. & Watson, I. A. Rules for identifying potentially reactive or promiscuous compounds. *J Med Chem* **55**, 9763-9772, doi:10.1021/jm301008n (2012).
12 Watson, I. A. *https://github.com/IanAWatson/Lilly-Medchem-Rules*.
13 SeeSAR (version 3.2) (2015).
14 Geertsma, E. R., Nik Mahmood, N. A., Schuurman-Wolters, G. K. & Poolman, B. Membrane reconstitution of ABC transporters and assays of translocator function. *Nat Protoc* **3**, 256-266, doi:10.1038/nprot.2007.519 (2008).
15 Karasawa, A. *et al.* Physicochemical factors controlling the activity and energy coupling of an ionic strength-gated ATP-binding cassette (ABC) transporter. *J Biol Chem* **288**, 29862-29871, doi:10.1074/jbc.M113.499327 (2013).
16 Karasawa, A. *et al.* Cystathionine beta-synthase (CBS) domains 1 and 2 fulfill different roles in ionic strength sensing of the ATP-binding cassette (ABC) transporter OpuA. *J Biol Chem* **286**, 37280-37291, doi:10.1074/jbc.M111.284059 (2011).
17 Souza, P. C. T. *et al.* Martini 3: a general purpose force field for coarse-grained molecular dynamics. *Nat Methods* **18**, 382-388, doi:10.1038/s41592-021-01098-3 (2021).
18 Kroon P C *et al.* Martinize2 and Vermouth: Unified Framework for Topology Generation. *Elife* **12**, doi:https://doi.org/10.7554/eLife.90627.1 (2023).
19 Setyawati, I. *et al.* In vitro reconstitution of dynamically interacting integral membrane subunits of energy-coupling factor transporters. *Elife* **9** (2020).
20 Faustino, I. *et al.* Membrane mediated toppling mechanism of the folate energy coupling factor transporter. *Nat Commun* **11**, 1763, doi:10.1038/s41467-020-15554-9 (2020).
21 Alessandri, R. *et al.* Martini 3 Coarse-Grained Force Field: Small Molecules. *Adv. Theory Simul.* **5**, 1-19 (2022).
22 Alessandri, R., Thallmair, S., Gil Herrero, C., Mera-Adasme, R., Marrink, S. J., and Souza, P. C. T.,. *A practical introduction to Martini 3 and its application to protein-ligand binding simulations,*. (AIP Publishing LLC, 2023).
23 L., J. W., Chandrasekhar J., Madura J. D., Impey R. W. & L., K. M. Comparison of simple potential functions for simulating liquid water. *The journal of Chemical Physics* **79**, 926–935, doi:doi.org/10.1063/1.445869 (1983).
24 Jorgensen, W. L. & Tirado-Rives, J. Potential energy functions for atomic-level simulations of water and organic and biomolecular systems. *Proc Natl Acad Sci U S A* **102**, 6665-6670, doi:10.1073/pnas.0408037102 (2005).
25 Dodda, L. S., Vilseck, J. Z., Tirado-Rives, J. & Jorgensen, W. L. 1.14*CM1A-LBCC: Localized Bond-Charge Corrected CM1A Charges for Condensed-Phase Simulations. *J Phys Chem B* **121**, 3864-3870, doi:10.1021/acs.jpcb.7b00272 (2017).
26 Dodda, L. S., Cabeza de Vaca, I., Tirado-Rives, J. & Jorgensen, W. L. LigParGen web server: an automatic OPLS-AA parameter generator for organic ligands. *Nucleic Acids Res* **45**, W331-W336, doi:10.1093/nar/gkx312 (2017).
27 Hilpert, C. *et al.* Facilitating CG Simulations with MAD: The MArtini Database Server. *J Chem Inf Model* **63**, 702-710, doi:10.1021/acs.jcim.2c01375 (2023).
28 Marrink, S. J., Risselada, H. J., Yefimov, S., Tieleman, D. P. & de Vries, A. H. The MARTINI force field: coarse grained model for biomolecular simulations. *J Phys Chem B* **111**, 7812-7824, doi:10.1021/jp071097f (2007).
29 Wassenaar, T. A., Ingolfsson, H. I., Bockmann, R. A., Tieleman, D. P. & Marrink, S. J. Computational Lipidomics with insane: A Versatile Tool for Generating Custom Membranes for Molecular Simulations. *J Chem Theory Comput* **11**, 2144-2155, doi:10.1021/acs.jctc.5b00209 (2015).
30 Carpenter, T. S. *et al.* Capturing Phase Behavior of Ternary Lipid Mixtures with a Refined Martini Coarse-Grained Force Field. *J Chem Theory Comput* **14**, 6050-6062, doi:10.1021/acs.jctc.8b00496 (2018).
31 Lomize, M. A., Pogozheva, I. D., Joo, H., Mosberg, H. I. & Lomize, A. L. OPM database and PPM web server: resources for positioning of proteins in membranes. *Nucleic Acids Res* **40**, D370-376, doi:10.1093/nar/gkr703 (2012).
32 de Jong Djurre H. , Baoukina S., I., I. H. & J., M. S. Martini straight: Boosting performance using a shorter cutoff and GPUs. *Computer Physics Communications* **199** (2016).
33 Souza, P. C. T., Limongelli, V., Wu, S., Marrink, S. J. & Monticelli, L. Perspectives on High-Throughput Ligand/Protein Docking With Martini MD Simulations. *Front Mol Biosci* **8**, 657222, doi:10.3389/fmolb.2021.657222 (2021).
34 G., B., D., D. & M., P. Canonical sampling through velocity rescaling. *The journal of Chemical Physics* **126**, doi:doi.org/10.1063/1.2408420 (2007).
35 Kjolbye, L. R. *et al.* Towards design of drugs and delivery systems with the Martini coarse-grained model. *QRB Discov* **3**, e19, doi:10.1017/qrd.2022.16 (2022).
36 Parrinello, M. & Rahman, A. Polymorphic transitions in single crystals: A new molecular dynamics method. *Journal of Applied Physics* **52**, 7182-7190 (1981).
37 Abraham, M. J. *et al.* GROMACS: High performance molecular simulations through multi-level parallelism from laptops to supercomputers. *Software X* **1-2** (2015).
38 Humphrey, W., Dalke, A. & Schulten, K. VMD: visual molecular dynamics. *J Mol Graph* **14**, 33-38, 27-38, doi:10.1016/0263-7855(96)00018-5 (1996).

39 Doudou, S., Burton, N. A. & Henchman, R. H. Standard Free Energy of Binding from a One-Dimensional Potential of Mean Force. *J Chem Theory Comput* **5**, 909-918, doi:10.1021/ct8002354 (2009).

40 Wassenaar, T. A., Pluhackova, K., Bockmann, R. A., Marrink, S. J. & Tieleman, D. P. Going Backward: A Flexible Geometric Approach to Reverse Transformation from Coarse Grained to Atomistic Models. *J Chem Theory Comput* **10**, 676-690, doi:10.1021/ct400617g (2014).

41 Wilkowska, K., Mruk, I., Furmanek-Blaszk, B. & Sektas, M. Low-level expression of the Type II restriction-modification system confers potent bacteriophage resistance in Escherichia coli. *DNA Res* **27**, doi:10.1093/dnares/dsaa003 (2020).
