# Supplementary material for: Identification of inhibitors targeting the energy-coupling factor (ECF) transporters
Source: Commun Biol. 2023 Nov 20;6:1182. doi: 10.1038/s42003-023-05555-x (PMC10662466; doi:10.1038/s42003-023-05555-x)
Supplement: Supplementary file 2 — Description of Additional Supplementary Files [file 42003_2023_5555_MOESM2_ESM.pdf]

Description of Additional Supplementary Files

File name: Supplementary Data 1
Description: The source data behind the graphs.
